# Investigating the underlying mechanisms of the ethanol extract of saussureae involucratae herba in anti-rheumatoid arthritis effect based on sphingolipidomics

**DOI:** 10.3389/fphar.2025.1549437

**Published:** 2025-06-18

**Authors:** Pingyuan Chi, Hairong Zhang, Yingjie Chen, Jianhua Xie, Yiming Ayixianmuguli, Caisheng Wu, Mingyuan Liu

**Affiliations:** ^1^ School of Pharmaceutical Sciences, Jiamusi University, Jiamusi, China; ^2^ Fujian Provincial Key Laboratory of Innovative Drug Target Research and State Key Laboratory of Cellular Stress Biology, School of Pharmaceutical Sciences, Xiamen University, Xiamen, China; ^3^ Xiamen Key Laboratory for Clinical Efficacy and Evidence-Based Research of Traditional Chinese Medicine, Xiamen University, Xiamen, China; ^4^ Drug Clinical Trial Institution, The First Affiliated Hospital, School of Medicine, Xiamen University, Xiamen, China; ^5^ Department of Food and Biological Engineering, Zhangzhou Institute of Technology, Zhangzhou, China; ^6^ School of Medical Sciences, Shihezi University, Shihezi, China

**Keywords:** saussureae involucratae herba, sphingolipidomics, rheumatoid arthritis, HPLC-MS/MS, S1P signaling pathway

## Abstract

**Introduction:**

Saussureae Involucratae Herba (SIH), a traditional Chinese Medicine, is clinically used in treating rheumatoid arthritis (RA). However, the anti-RA mechanisms of SIH remain unclear. Dysregulation of sphingolipid metabolism is related to the pathogenesis of RA. This study aims to investigate whether the regulation of sphingolipid metabolism is involved in the anti-RA effects of an ethanol extract of SIH (SIE).

**Methods:**

The collagen-induced arthritis (CIA) mouse model and LPS-stimulated RAW 264.7 cells were used. Targeted sphingolipidomics were employed to investigate the effects of SIE on the regulation of sphingolipid metabolism in CIA mice.

**Results:**

Results showed that SIE significantly reduced arthritis scores and the average thickness of the four paws (both *P* < 0.01) in CIA mice. Additionally, it improved histopathological manifestations (including synovial hyperplasia, inflammatory cell infiltration, cartilage and bone destruction) in the ankle joints of CIA mice, and inhibited bone erosion in the ankle and toe joints. In cell assays, SIE significantly decreased the protein levels of TNF-α and IL-6 (both *P* < 0.01) in LPS-stimulated RAW 264.7 cells. Mechanistically, SIE treatment normalized the concentration of seven sphingolipids in plasma and eight sphingolipids in spleen, which were identified as potential anti-RA targets of SIE. Meanwhile, SIE treatment significantly lowered the protein level of SphK1 and the content of S1P (both *P* < 0.01) in LPS-stimulated RAW 264.7 cells.

**Discussion:**

We, for the first time, found that SIE has anti-RA effects in CIA mice and that regulation of sphingolipid metabolism is involved in its anti-RA action. These findings provide pharmacological evidence for the use of SIH in managing RA and support the theory that targeting sphingolipid metabolism is a strategy for treating RA.

## 1 Introduction

Rheumatoid arthritis (RA) is a chronic autoimmune disease. Its typical clinical manifestations include symmetrical polyarthritis, often accompanied by pain, fatigue, joint dysfunction, and emotional disturbances, which can lead to disability and deformity (Zhang et al., 2022). In addition, compared to the general population, RA patients take a higher risk of developing osteoporosis, cardiovascular diseases, and cancer, with a comorbidity rate as high as 60% (Richards et al., 2015; Sparks, 2019). Among these, cardiovascular diseases increase the mortality rate of RA patients by about 60% (Chen J.-L. et al., 2020). RA is observed globally across various regions and ethnic groups, with a global prevalence of approximately 1.0% (Zhang et al., 2022). Currently, the main medications used to treat RA include disease-modifying antirheumatic drugs (DMARDs, such as methotrexate and leflunomide), nonsteroidal anti-inflammatory drugs (NSAIDs, such as aspirin, indomethacin, and ibuprofen), and glucocorticoids (such as triamcinolone, dexamethasone palmitate, and prednisolone). Although these treatments demonstrate some clinical efficacy, they often come with adverse reactions (Burmester and Pope, 2017), and some patients do not respond well to the existing treatments (Chen Y.-J. et al., 2020), highlighting the urgent need to develop new therapies.

Traditional Chinese medicine, including ethnic medicines, possesses multi-target characteristics, making them advantageous for treating complex diseases. Saussureae Involucratae Herba (SIH) is the dried aerial part of the plant *Saussurea involucrata* (Kar. et Kir.) Sch.-Bip. from the Asteraceae family, mainly distributed in the Tianshan and Altai Mountains of Xinjiang, China (Hu et al., 2023). As an ethnic medicine, SIH has a long history of use in traditional medicine, with records in classical Tibetan medical books the *Yue Wang Yao Zhen* (Somaratsa, Moon king Medical Manual) and the *Si Bu Yi Dian* (Gyud-Zhi, The Four Medical Tantras) as early as the 8th century (Xu, 2019). According to the 2020 edition of the “Pharmacopoeia of the People’s Republic of China,” SIH has the effects of warming kidney and tonifying Yang, dispelling wind and dampness, and promoting blood circulation, commonly used for diseases including wind-cold-damp arthralgia and RA (Committee, 2020). Currently, SIH is the main active ingredient of some proprietary Chinese medicines use for treating RA, such as Xuelian injection and Xuelian oral liquid (Cong et al., 2022; Yan et al., 2023). However, the specific mechanisms of SIH in treating RA remain unclear, which hinders its further development and broader application in clinical settings.

Previous studies have shown that sphingolipid metabolism may play a role in RA pathogenesis (Qu et al., 2018). The pathogenesis of rheumatoid arthritis (RA) is complex, with the SphK1/S1P signaling pathway playing a crucial role. Studies have shown that increased SphK1 activity leads to excessive production of S1P, thereby promoting inflammatory responses and joint damage in RA (Lai et al., 2008; Wang et al., 2021). SPLs are a class of complex lipid molecules primarily found in cell membranes. SPLs play essential roles in cell membrane structure and function, participating in signal transduction, molecular transport, apoptosis, cell proliferation, and other physiological processes (Hannun and Obeid, 2008). Sphingolipid metabolism affects the function of immune cells (such as T cells and macrophages) and fibroblast-like synovial cells (FLS) in RA patients (RA-FLS) (Guo et al., 2018; Komatsu and Takayanagi, 2022). Cer, SM and S1P are three important sphingolipid metabolic products (Hannun and Obeid, 2017). Cer and SM can be interconverted, and Sph and S1P can be interconverted under the catalysis of SphK1. Cer can induce apoptosis of RA-FLS cells and inhibit cell invasion and migration (Yang M. et al., 2022), whereas S1P can promote RA-FLS survival and proliferation (Takeshita et al., 2012). It has been reported that SphK1 is one of the therapeutic targets for RA (Baker et al., 2010). In a TNF-α-induced arthritis mouse model, knocking out SphK1 inhibited synovitis and bone erosion. These findings indicate that sphingolipid metabolism plays an important role in the pathogenesis of RA.

Sphingolipids (SPLs) are bioactive lipids that play crucial roles in cell signaling, inflammatory responses, and immune functions (Hannun and Obeid, 2017). Increasing evidence suggests that dysregulation of sphingolipid metabolism is related to the pathogenesis of RA (Hanaoka et al., 2017; Koh et al., 2022), and certain key compounds in sphingolipid metabolism, such as sphingosine 1 phosphate (S1P), are considered as new targets for RA treatment (Hu et al., 2010). Compounds in SIH such as chlorogenic acid (Ping et al., 2024) and quercetin (Momchilova et al., 2022) have been reported to regulate sphingolipid metabolism in cardio-myoblast and cancer cell lines, respectively. Additionally, luteolin (Navone et al., 2023) and apigenin (Zhang et al., 2015) can regulate components in the sphingolipid metabolic pathway, such as sphingosine kinase 1 (SphK1), S1P, and ceramide (Cer). Therefore, we hypothesize that the therapeutic effects of SIH on RA may be partially achieved through the regulation of sphingolipid metabolism. The purpose of this study was to determine whether the therapeutic effect of SIH on RA is mediated by the regulation of key molecules of sphingolipid metabolic pathway (such as ceramide, sphingosine-1-phosphate, etc.)

## 2 Results

### 2.1 An extract of SIH (SIE) alleviated arthritis symptoms in CIA mice

**FIGURE 1:**
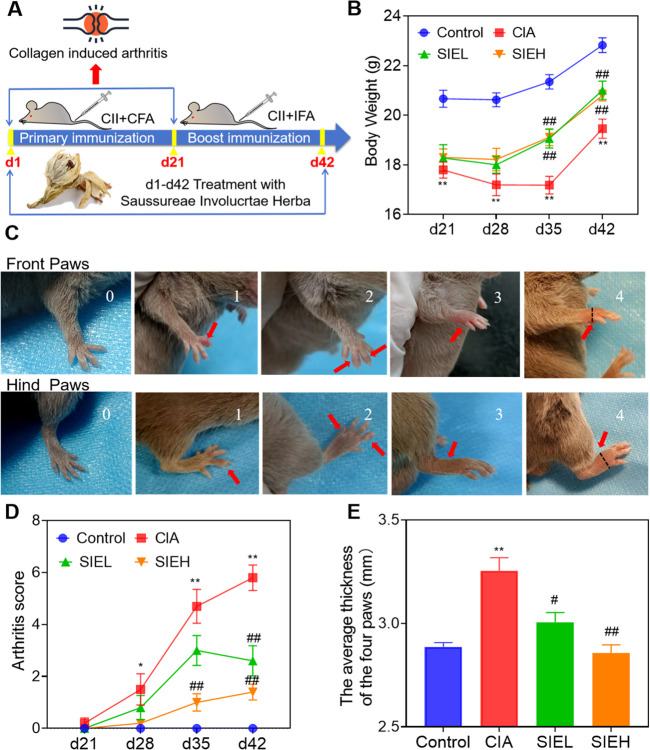
Effects of SIE on clinical symptoms of mice with collagen induced arthritis. **(A)** Establishment of the CIA model and administration methods; **(B)** Body weight of each group; **(C)** Arthritis scoring criteria: The upper panel represents the forepaws, while the lower panel represents the hind paws. The four paws of mice were scored respectively. Scores of 0, 1, 2, 3, and 4 correspond to the scores of the mice’s paws shown in the images. The maximum cumulative arthritis score for the four paws of a mouse is 16 points. The black dashed line indicates the position where the caliper measures the thickness of the paws (on the far-right image). The four paw thicknesses of mice were measured respectively. **(D)** Arthritis score of each group. **(E)** The average thickness of the four paws of the mice. The paw thickness was measured on day 42. Group comparisons were conducted using Dunnett’s t-test and Mann-Whitney U test. Data are presented as mean ± standard error of mean (n = 10 per group). Compared to the control group, *P < 0.05, **P < 0.01; compared to the CIA group, ^#^ *P* < 0.05, ^##^ *P* < 0.01.

Collagen-induced arthritis (CIA) DBA-1J mice serve as a classic RA animal model, sharing similar immunological and pathological features with human RA (Brand et al., 2007). Therefore, we selected this mouse model to study the anti-RA effects of SIE (an ethanol extract of SIH). As shown in Figure 1A, we evaluated the therapeutic effect of SIE on CIA by constructing the mouse CIA model. As shown in Figure 1B, from day 21 to day 42, the body weight of the control group mice continued to increase under normal feeding conditions, while the body weight of the CIA model group mice decreased from day 21 to day 35 after modeling. By day 42, the weight gain of mice in the CIA group was significantly less than that in the control group. After SIE treatment, the low-dose group of SIE (SIEL) and high-dose group (SIEH) showed an improvement in the reduced weight gain caused by disease progression compared to the CIA group mice at days 35 and 42.

**FIGURE 2:**
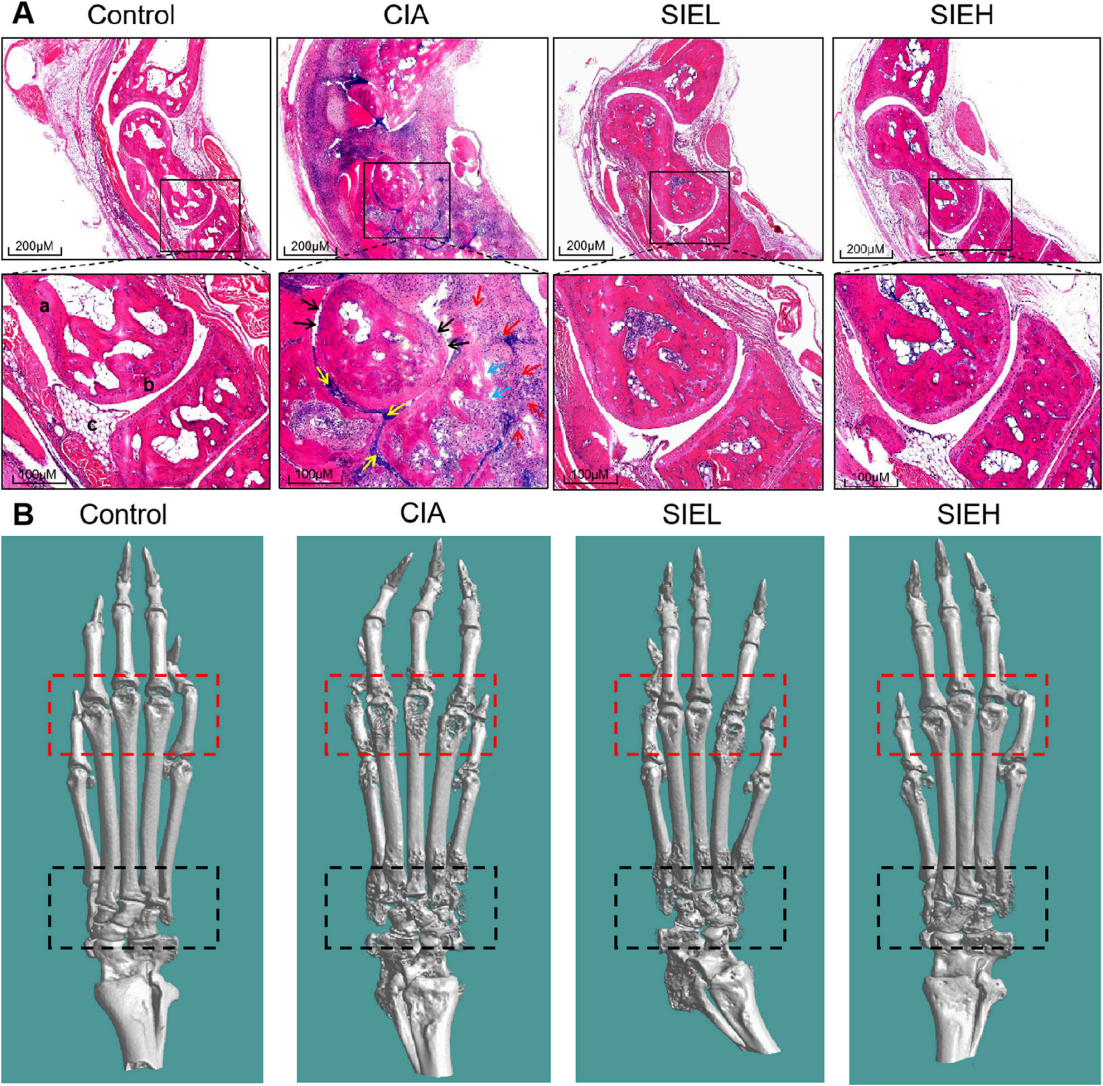
**(A)** Representative images of hematoxylin and eosin (H&E)-stained ankle joint tissues from mice in each group. Scale bars: 200 μm (upper panel) and 100 μm (lower panel). a: bone, b: articular cartilage, c: synovium; black arrow (↑): cartilage destruction, blue arrow (↑): bone destruction, red arrow (↑): inflammatory cell infiltration, yellow arrow (↑): synovial hyperplasia. **(B)** Representative micro-computed tomography (Micro CT) images of mouse hind limbs. The red dashed box outlines the toe joints, while the black dashed box outlines the ankle joint.

As shown in Figure 1C, arthritis scores were evaluated based on a 0–4 scoring standard for the forepaws and hind paws of the mice (Alabarse et al., 2018). As shown in Figure 1D, starting on day 28, the arthritis scores of mice in the CIA group were significantly higher than those in the control group, reaching the highest value on day 42. After SIE treatment, the arthritis scores of the SIEL group mice were significantly lower than those of the CIA group on day 42, and the scores of the SIEH group mice were significantly lower than those of the CIA group on days 35 and 42. As shown in Figure 1E, on day 42, the paw thickness of the CIA group mice was significantly higher than that of the control group. After treatment, the paw thickness of mice in the SIEL and SIEH groups was significantly lower than that in the CIA group. As shown in Figure 2A, HE staining results indicated that, compared with the control group, the CIA group mice had blurred joint contours, reduced joint cavity space, significant synovial hyperplasia, inflammatory cell infiltration, and severe cartilage and bone structure damage. After treatment, these pathological changes in the SIEL and SIEH group mice were improved. Micro CT results (Figure 2B) indicated that compared to the control group, the CIA group mice had rough joint surfaces, and cavities appeared in the ankle and toe joints, indicating severe bone erosion. After treatment, the bone erosion in the ankle and toe joints of both the SIEL and SIEH groups was significantly improved. The *in vivo* data demonstrate that SIE effectively inhibited disease progression without causing observable toxicities and adverse reactions in CIA mice. The serum cardiac, hepatic, and renal toxicity indicators in mice treated with SIEH did not significantly differ from those in the control group (Supplementary Figure S2).

### 2.2 Exploring the impact of SIE on sphingolipid metabolism in CIA mice based on targeted sphingolipidomics

#### 2.2.1 Targeted sphingolipidomics analysis in plasma and spleen of mice

**FIGURE 3:**
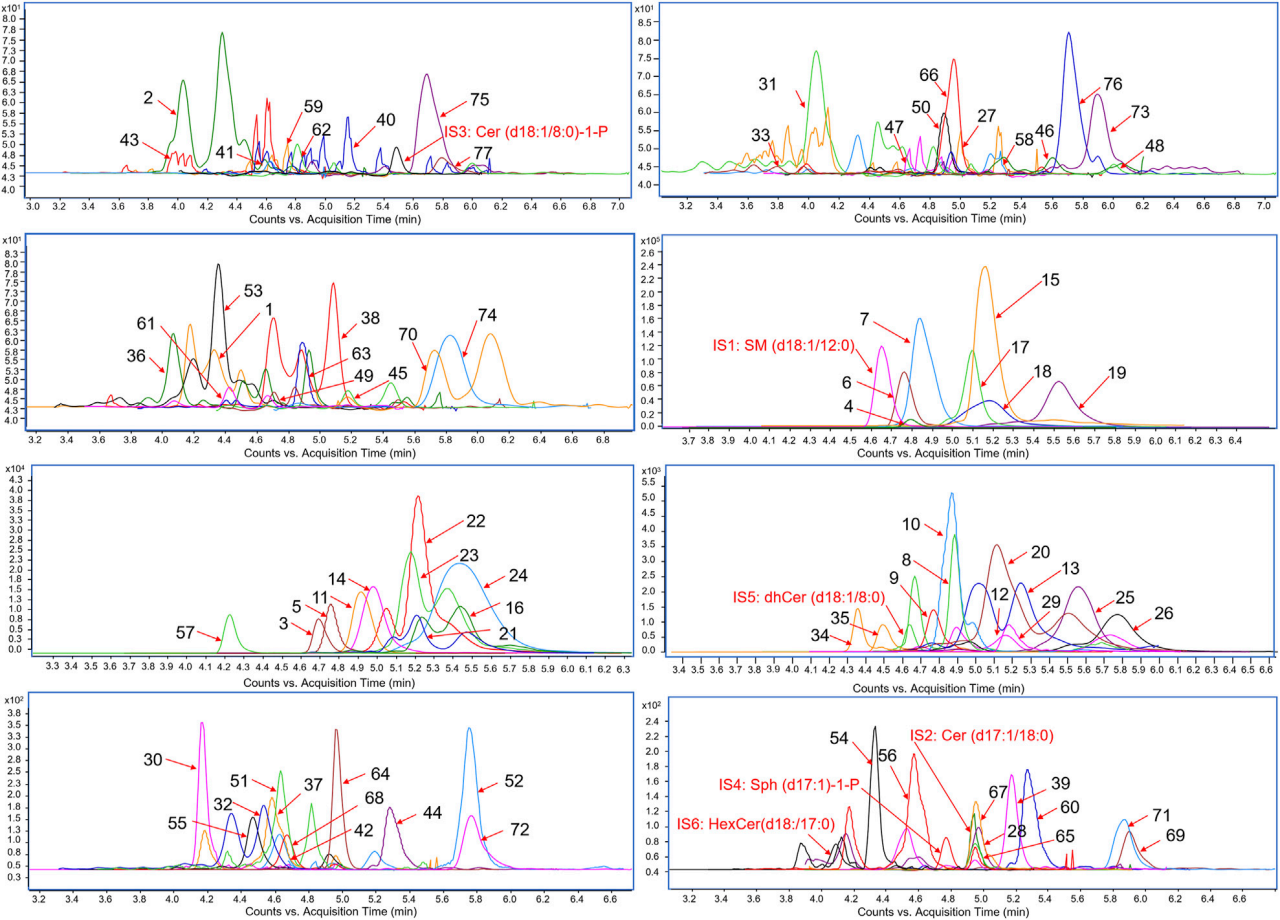
Chromatograms of 77 sphingolipids (SPLs) and 6 internal standard (IS) compounds.

**FIGURE 4:**
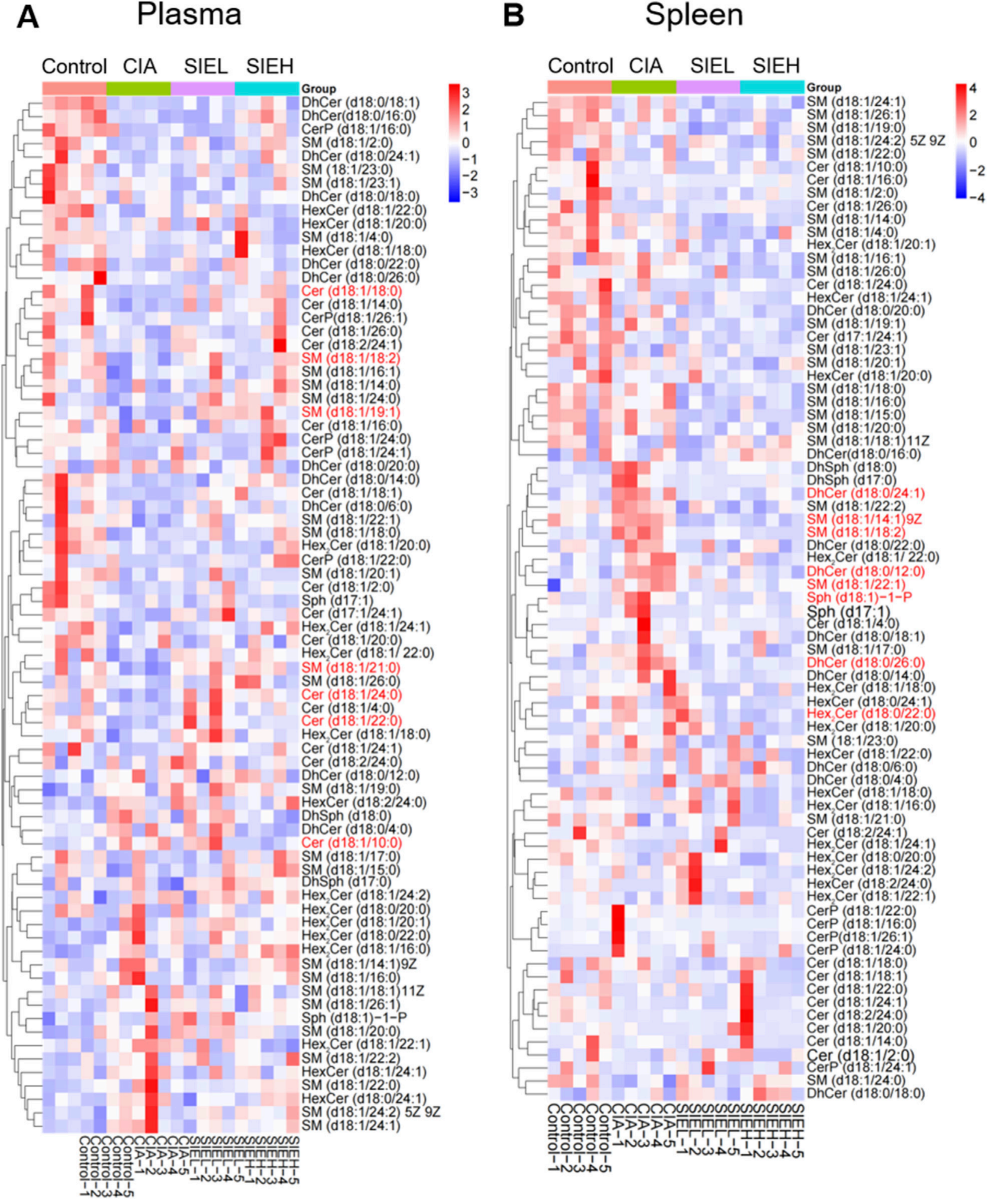
Heatmaps of differential metabolities in plasma **(A)** and spleen **(B)**. The metabolites highlighted in red represent potential anti-RA biomarkers of SIE that were screened out in subsequent experiments.

To investigate the impact of SIE on sphingolipid metabolism in the plasma and spleen of CIA mice, we used targeted sphingolipidomics analysis based on HPLC-MS/MS. As shown in Figure 3, under the positive ion mode of the electro-spray ionization (ESI) source, we detected a total of 77 SPLs, including 8 categories: sphingomyelin (SM), Cer, dihydroceramide (DhCer), ceramide-1-phosphate (Cer-1-P), sphingosine (Sph), dihydrosphingosine (DhSph), S1P (also known as Sph-1-P), and hexosylceramide (HexCer), along with 6 internal standards (IS). The heatmap (Figure 4) of the 77 SPLs shows the differences in their relative contents among the four groups of mice. The relative content of the 77 SPLs was used to establish principal component analysis (PCA) and orthogonal partial least squares discriminant analysis (OPLS-DA) models.

**FIGURE 5:**
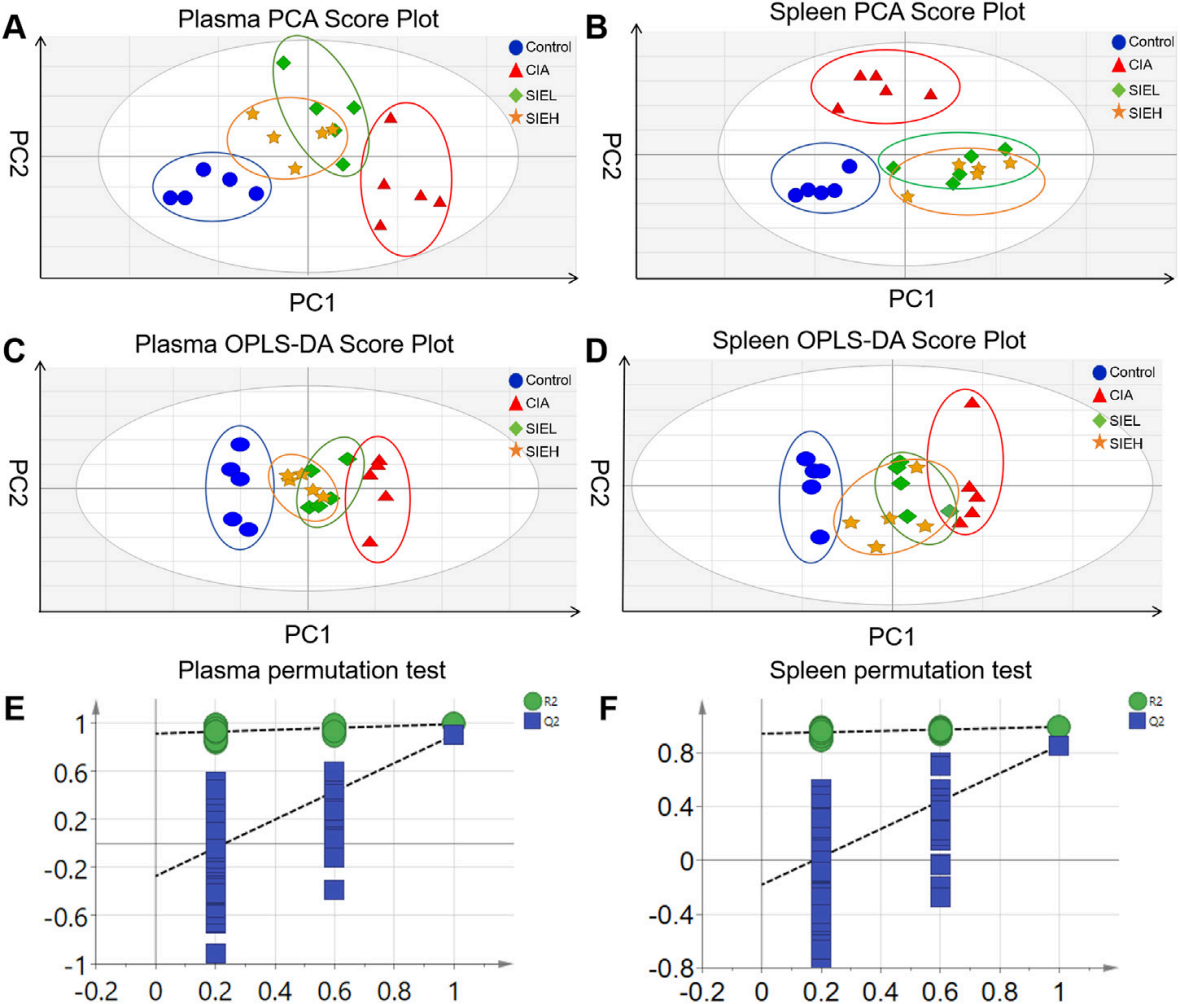
Principal component analysis (PCA) score plots of plasma **(A)** and spleen **(B)**; Orthogonal partial least squares discriminant analysis (OPLS-DA) score plots of plasma **(C)** and spleen **(D)**; OPLS-DA model permutation test plots of plasma **(E)** and spleen **(F)**.

Unsupervised PCA models were used to visualize metabolic differences between groups. As shown in Figures 5A,B, the PCA results revealed clear separation between the plasma and spleen samples of the control group mice and the CIA group mice. The SIEL and SIEH groups’ mice samples were situated between the control group mice and CIA group mice, showing a trend towards the control group mice. This indicates that CIA induces sphingolipid metabolic disorders, and SIE can regulate these disorders induced by CIA.

To identify potential biomarkers for the anti-RA effects of SIE, supervised OPLS-DA was employed to maximize the differences between groups. The parameters related to the reliability of the OPLS-DA model were as follows: R^2^X (cum) = 0.475, R^2^Y (cum) = 0.992, Q^2^ (cum) = 0.903 (plasma); R^2^X (cum) = 0.387, R^2^Y (cum) = 0.995, Q^2^ (cum) = 0.82 (spleen). Generally, Q^2^ > 0.5 indicates a reliable model (Chen et al., 2024). The Q^2^ values of the samples suggest that the OPLS-DA model is reliable. As shown in Figures 5C,D, the OPLS-DA results display good differentiation and clear clustering trends between the control and CIA groups, as well as between the CIA and SIEL/SIEH groups in both plasma and spleen.

To prevent overfitting during model construction, permutation tests were performed to ensure the validity of the models. The parameters related to the validity of the permutation tests were as follows: R^2^ = 0.92, Q^2^ = −0.22 (plasma); R^2^ = 0.948, Q^2^ = −0.14 (spleen). Generally, a Q^2^ intercept <0 indicates a reliable model (Yang et al., 2024). As shown in Figures 5E,F, the permutation test results revealed that all arranged Q^2^ values were lower than the original values, indicating that there was no overfitting in the OPLS-DA models, and they had good stability and predictive ability.

These results suggest that SIE exerts anti-RA effects by regulating sphingolipid metabolism.

#### 2.2.2 Screening of potential biomarkers in plasma and spleen of mice

**FIGURE 6:**
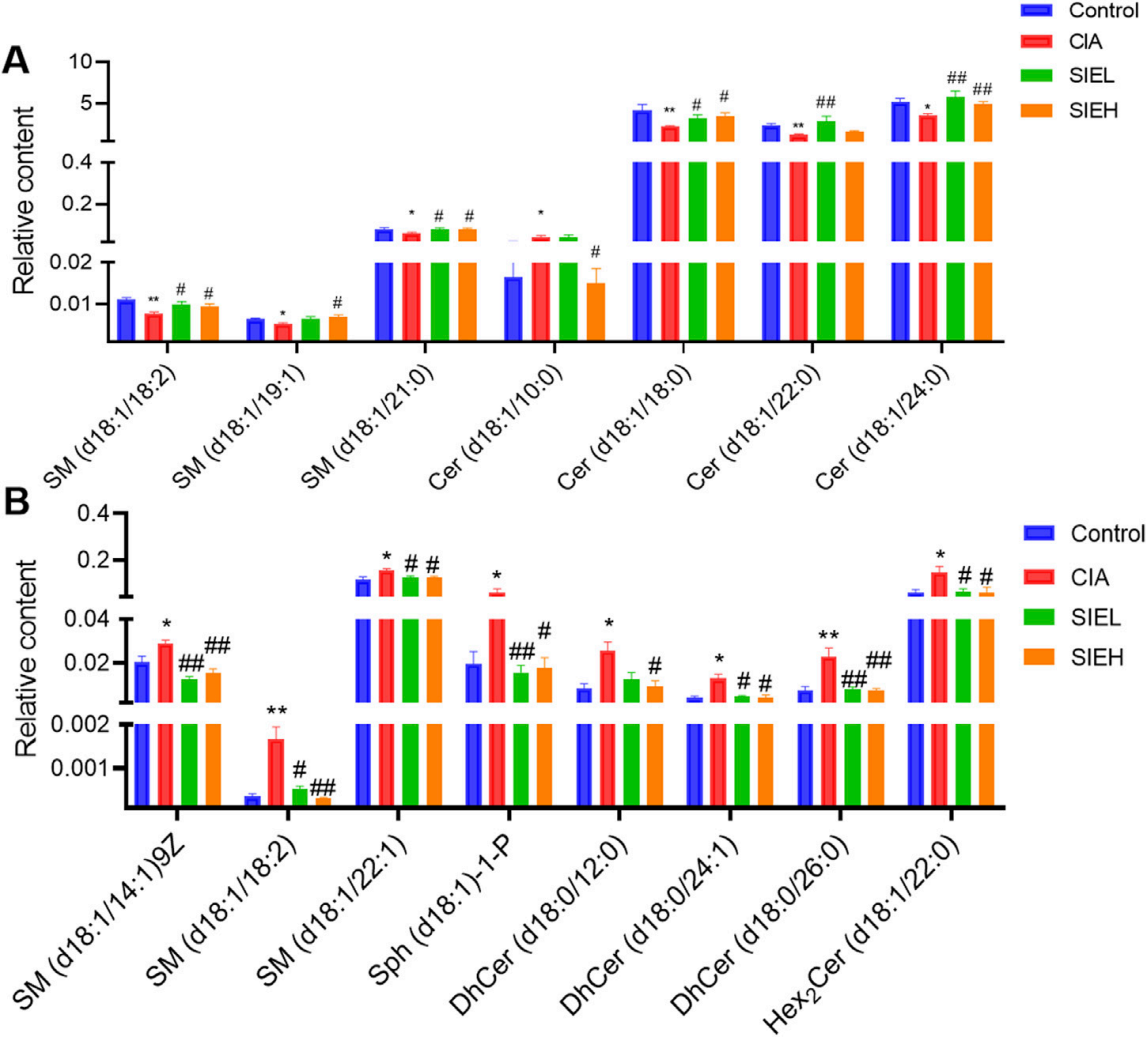
Relative content of potential biomarkers related to RA and SIE anti-RA in plasma **(A)** and spleen **(B)**. Group comparisons were performed using Dunnett’s t-test and Mann-Whitney U test. Data are presented as mean ± standard error of mean (n = 5 per group). Compared to the control group, *P < 0.05, **P < 0.01; compared to the CIA group, ^#^ *P* < 0.05, ^##^ *P* < 0.01.

The variable importance in projection (VIP) values of the metabolites were calculated using the OPLS-DA model, and potential biomarkers in plasma and spleen were screened with VIP >1 and P < 0.05. As shown in Figure 6A, seven potential biomarkers were identified in the plasma, including SPLs in two categories: SM and Cer. Compared with the control group, the levels of SM (d18:1/18:2, downregulated), SM (d18:1/19:1, downregulated), SM (d18:1/21:0, downregulated), Cer (d18:1/10:0, upregulated), Cer (d18:1/18:0, downregulated), Cer (d18:1/22:0, downregulated), and Cer (d18:1/24:0, downregulated) in the CIA group showed significant changes. After SIE treatment, these seven potential biomarkers in CIA mice showed a reversal in levels compared with the model group, either with low or high doses of SIE.

As shown in Figure 6B, eight potential biomarkers were identified in the spleen, including SPLs from four categories: SM, Sph-1-P, DhCer, and HexCer. Compared with the control group, the levels of SM (d18:1/14:1)9Z, SM (d18:1/18:2), SM (d18:1/22:1), Sph (d18:1)-1-P, DhCer (d18:0/12:0), DhCer (d18:0/24:1), DhCer (d18:0/26:0), and Hex_2_Cer (d18:1/22:0) in the CIA group were significantly upregulated. After SIE treatment, these eight potential biomarkers showed a reversal in levels with either low or high doses of SIE.

### 2.3 Effects of SIE on inflammatory cytokines and the SphK1/S1P signaling pathway in LPS-stimulated RAW 264.7 cells

#### 2.3.1 CCK8 assay

**FIGURE 7:**
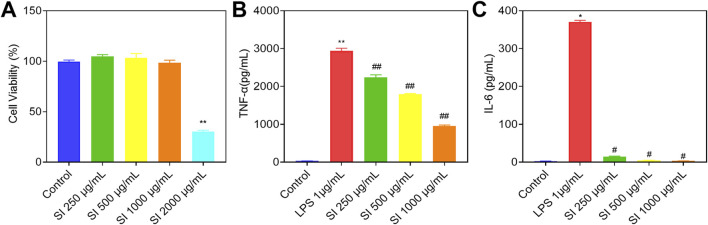
Effects of SIE on the protein levels of inflammatory factors. **(A)** Cell viability; **(B)** The protein level of TNF-α in cell supernatant among the five groups; **(C)** The protein level of IL-6 in cell supernatant among the five groups. Group comparisons were performed using Dunnett’s t-test and Mann-Whitney U test. Data are expressed as mean ± standard error of mean (n = 3 per group). Compared to the control group, *P < 0.05, **P < 0.01; compared to the CIA group, ^#^ *P* < 0.05, ^##^ *P* < 0.01.

To explore the non-toxic concentrations of SIE in a cellular model, we first used the CCK-8 assay to detect the effects of different concentrations of SIE on the viability of RAW 264.7 cells. As shown in Figure 7A, results showed that SIE had no effect on the viability of RAW 264.7 cells at concentrations of 250 μg/mL, 500 μg/mL, and 1,000 μg/mL. However, at a concentration of 2000 μg/mL, cell viability significantly decreased. Therefore, 250 μg/mL, 500 μg/mL, and 1,000 μg/mL were selected as non-toxic concentrations used for subsequent studies.

#### 2.3.2 Effects of SIE on inflammatory cytokines in LPS-stimulated RAW 264.7 cells

The protein levels of tumor necrosis factor-α (TNF-α) and interleukin 6 (IL-6) in each group of cells were detected using enzyme-linked immunosorbent assay (ELISA) (Zuo et al., 2024). As shown in Figures 7B,C, the protein levels of TNF-α and IL-6 significantly increased after LPS stimulation for 24 h in RAW 264.7 cells. After treatment with low, medium, and high doses of SIE for 24 h, the protein levels of TNF-α and IL-6 significantly decreased in a dose-dependent manner. These results suggest that SIE can inhibit the secretion of inflammatory cytokines in the cellular model.

**FIGURE 8:**
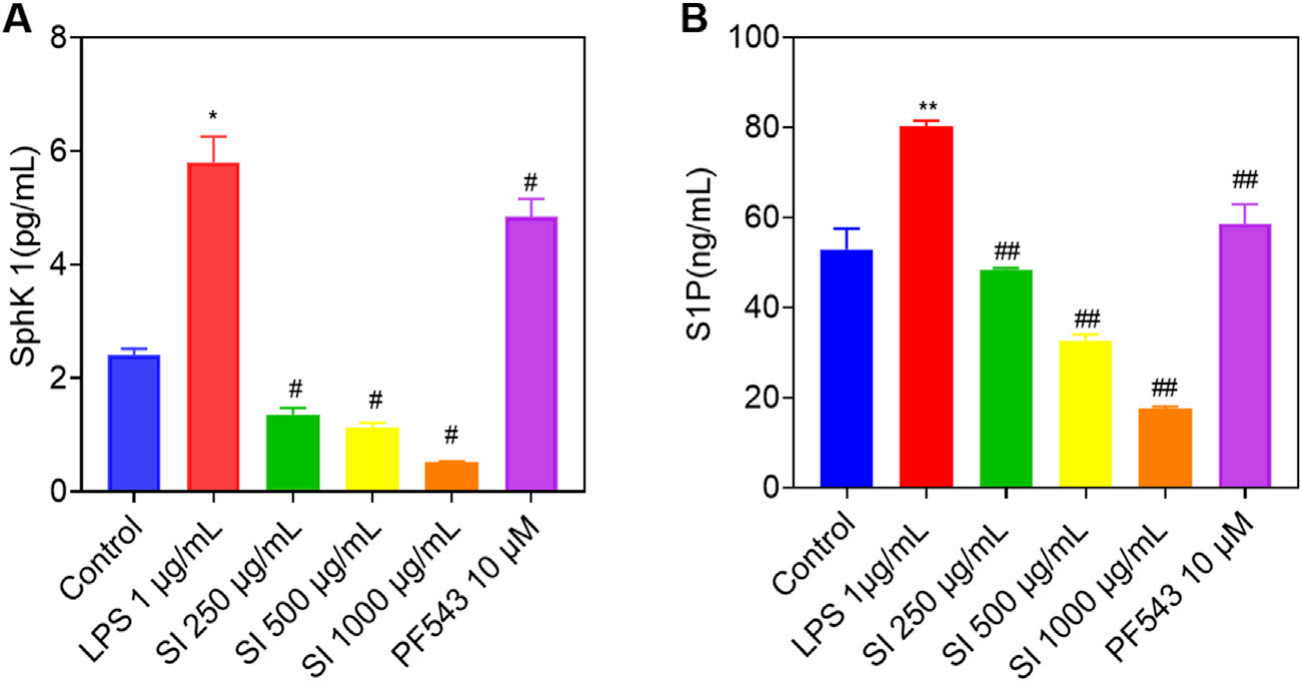
Effects of SIE on the protein levels of SphK1 and the content of S1P. **(A)** The protein level of SphK1 in cell supernatant among the five groups; **(B)** The content of S1P in cell supernatant among the five groups. Group comparisons were performed using Dunnett’s t-test and Mann-Whitney U test. Data are expressed as mean ± standard error of mean (n = 3 per group). Compared to the control group, *P < 0.05, **P < 0.01; compared to the CIA group, ^#^ *P* < 0.05, ^##^ *P* < 0.01.

The protein level of sphingosine kinase 1 (SphK1) and the content of sphingosine 1 phosphate (S1P) in various groups of cells were detected using ELISA. As shown in Figures 8A,B, the protein level of SphK1 and the content of S1P significantly increased after stimulating with LPS for 24 h in RAW 264.7 cells. After treating with low, medium, and high doses of SIE for 24 h, the protein level of SphK1 and the content of S1P decreased significantly in a dose-dependent manner. Similarly, the protein level of SphK1 and the content of S1P also significantly decreased after administering with PF543 (positive control, SphK1 inhibitor) for 24 h (Cheresh et al., 2020). These findings suggest that SIE’s inhibition of SphK1/S1P is involved in SIE’s anti-inflammatory effect.

## 3 Discussion

Previous studies have showed that the ethanol extract of SIH has anti-RA effects in CIA and adjuvant-induced arthritis (AIA) rat models (Han et al., 2016; Xu et al., 2016). In the present study, we found that SIE had anti-RA effects in the CIA mouse model, including alleviating paw thickness (Figure 1E), reducing inflammatory cell infiltration in the joints (Figure 2A), and inhibiting bone erosion (Figure 2B). Moreover, SIE ameliorated weight loss caused by the disease (Figure 1B) and SIEH exhibited no toxicities in mice (Supplementary Figure S2). The serum biochemical parameters of liver, kidney, and cardiac toxicity in the SIEH group of mice were comparable to those in the normal group (Supplementary Figure S2). In LPS-stimulated RAW 264.7 cells, SIE inhibited the release of inflammatory cytokines TNF-α and IL-6 (Figures 7B,C). These findings support the safety and efficacy of SIE in treating RA.

As mentioned in the introduction, the aim of this study is to explore the potential anti-RA mechanisms of SIE from the perspective of sphingolipid metabolism. Both *in vivo* and *in vitro* models to analyze the regulatory effects of SIE on sphingolipid metabolism. A bovine type II collagen (CII)-induced collagen-induced arthritis (CIA) mouse model was used to evaluate the therapeutic effects of SIE. High performance liquid chromatography-tandem mass spectrometry (HPLC-MS/MS) combined with multivariate statistical analysis (Yang et al., 2024) was employed to study SIE’s impact on sphingolipid metabolism in CIA mice. Additionally, lipopolysaccharide (LPS)-stimulated RAW 264.7 macrophage model was used as an *in vitro* inflammation model to examine the direct effects of SIE on the production of inflammatory factors and the activity of the SphK1/S1P signaling pathway, which is closely related to the pathophysiological process of RA and sphingolipid metabolism (Wang et al., 2021).

**FIGURE 9:**
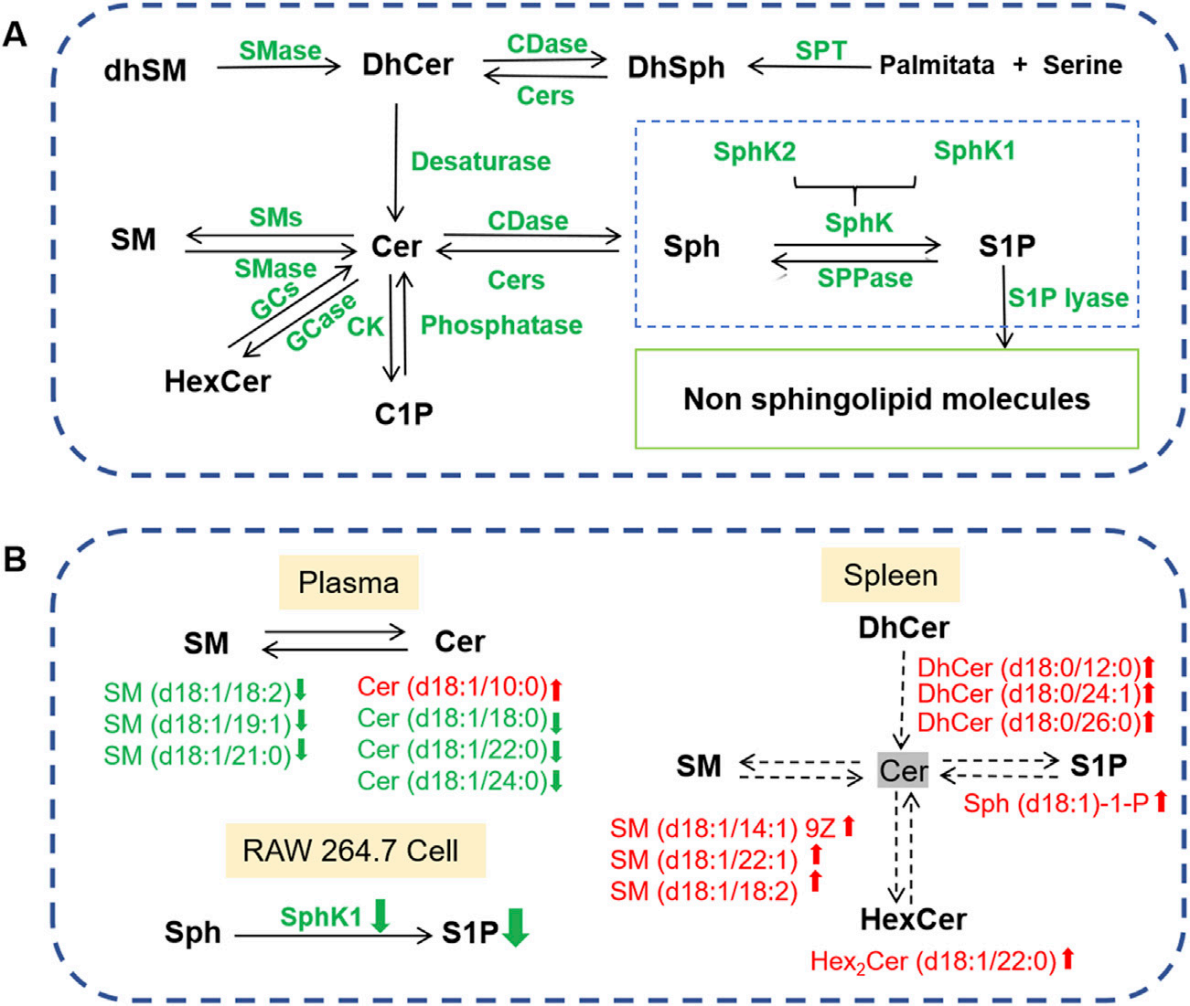
**(A)** Sphingolipid metabolism network diagram. SPT, serine palmitoyltransferase; CDase, ceramidase; Cers, ceramide synthetase; CK, ceramide kinase; GCase, glucosyl ceramidase; GCS, glucosylceramide synthase; SphK, sphingosine kinase; SMase, sphingomyelinase; SMS, sphingomyelin synthase; SPPase, sphingosine phosphate phosphatase. **(B)** Effects of SIE on sphingolipid metabolism in the plasma and spleen of CIA mice, as well as the supernatant of lipopolysaccharide (LPS)-stimulated RAW 264.7 cells.

As shown in Figure 9, the SPLs metabolic network is highly complex, connecting the metabolism of many SPLs compounds, such as SM, Cer, DhCer, HexCer, Cer-1-P, Sph, DhSph, S1P, and others. Each SPLs compound shows complex patterns of change in the serum, cells, and tissues of RA patients, encompassing various subtypes. For example, some specific SPLs molecule levels in the serum of RA patients and AIA rats are higher, while some are lower compared to healthy individuals and normal rats (Luan et al., 2023). This may be due to structural differences between subtypes, leading to differences in their distribution, biological functions, and metabolic pathways in cell membranes. RA-related oxidative stress may cause certain SM subtypes to be more easily oxidized or degraded, while others may remain relatively stable. In this study, we identified two classes (seven) SPLs biomarkers related to the anti-RA effect of SIE in mouse plasma and four classes (eight) biomarkers in the spleen (Figures 6). SIE reversed the disease-induced dysregulation of 15 SPLs compounds in plasma and spleen of CIA mice (Figures 6). In LPS-stimulated RAW 264.7 cells, SIE inhibited the expression of SphK1 and S1P (Figures 8). It is noteworthy that the SphK1/S1P signaling pathway plays a significant role in the pathogenesis of rheumatoid arthritis (RA). Previous studies have established RA models indicating that the expression levels of SphK1 and S1P are significantly increased, which is consistent with the results of this study and further supports their importance in RA (Hammad et al., 2007; Sun et al., 2020). In future studies, it will be necessary to examine whether SIE inhibits the expression of SphK1 and S1P in the synovial tissue of CIA mice; the sphingolipid metabolism in the synovial tissue of CIA mice should also be investigated.

The complexity of sphingolipid metabolism is reflected in the fact that the same sphingolipid can exhibit different changes in level across different tissues or organs under disease conditions. For instance, during RA progression, six sphingolipids such as SM(d18:1/18:0) and SM(d18:1/20:0) are downregulated in serum (Qu et al., 2018) but upregulated in synovial tissue (Kosinska et al., 2014). In our study, we also observed a similar phenomenon. In Figure 6, compared to the normal group, the trend of change in SM(d18:1/18:2) is opposite in the plasma (decrease) and spleen tissue (increase) for the model group mice. This may be attributed to the distinct microenvironmental changes in different tissues or organs, such as serum and spleen, throughout RA progression. These specific sphingolipids may play different physiological roles in serum and spleen, leading to differing trends of change in disease settings. Furthermore, the roles of specific sphingolipids in the pathological process of RA remain unknown, which warrants further investigation in the future.

According to the 2020 edition of the “Pharmacopoeia of the People’s Republic of China,” SIH is used by decoction or soaking in alcohol. Both water and ethanol extracts of SIH have been reported to have anti-RA effects in CIA rats. In our previous studies, UPLC-HRMS was used for a preliminary analysis of the components of ethanol and water extracts of SIH, identifying 151 components in a ethanol extract based on molecular weight and fragmentation patterns, as detailed in the Supplementary Table S1 (Yang L. et al., 2022). Our network pharmacology analysis identified seven effective anti-RA components: umbelliferone, apigenin, hispidulin, chlorogenic acid, arctigenin, syringin, and quercetin. These compounds have all been reported to have anti-RA activity (Song et al., 2010; Chauhan et al., 2011; Liu et al., 2015; Kim et al., 2019; Li et al., 2019; Yuan et al., 2020; Cai et al., 2022). We also found that the content (Yang L. et al., 2022) and bioavailability (Yang L. et al., 2022) of effective components in the ethanol extract of SIH are higher than in the water extract. Therefore, in this experiment, we prepared SIE using a 65% ethanol reflux extraction method and detected the content of three effective components, as detailed in the Supplementary Figure S1 and Supplementary Table S2. The content of these three components is close to that in our previous studies on the ethanol extract. As mentioned in the introduction, compounds such as chlorogenic acid (Ping et al., 2024), quercetin (Momchilova et al., 2022), luteolin (Navone et al., 2023), and apigenin (Zhang et al., 2015) can regulate sphingolipid metabolism or key sphingolipid metabolic products in cell models. Whether these four compounds have the effect of regulating sphingolipid metabolism in CIA mice deserves further study.

RA-FLS are commonly used in the study of the mechanisms of anti-RA drugs. RA-FLS are characterized by excessive proliferation and apoptosis resistance. They secrete inflammatory cytokines (Chen et al., 2021) and the osteoclastogenesis-promoting factor RANKL (Tunyogi-Csapo et al., 2008). As mentioned earlier, Cer can induce apoptosis in RA-FLS cells and inhibit cell invasion and migration, while S1P can promote the survival and proliferation of RA-FLS cells (Takeshita et al., 2012; Yang M. et al., 2022). Whether SIE can inhibit the expression of SphK1 and S1P in RA-FLS cells will also be investigated.

## 4 Materials and methods

### 4.1 Chemicals and reagents

IS: SM (d18:1/12:0), batch number: 860583P; Cer (d17:1/18:0), batch number: 860646P; DhCer (d18:0/8:0), batch number: 860626P; HexCer (d18:1/17:0), batch number: 860569P was purchased from Avanti Polar Lipids (Alabama, USA). C1P (d18:1/8:0), batch number: 62547; Sph (d17:1), batch number: 10007902 was purchased from Cayman Chemical (Michigan, USA). Methanol (chromatographic grade), isopropanol (chromatographic grade), methyl tert-butyl ether (analytical grade), formic acid, and ammonium acetate were purchased from Shanghai Macklin Biochemical Co., Ltd. (Shanghai, China). Acetonitrile (chromatographic grade) was purchased from Sigma-Aldrich (Shanghai) Trading Co., Ltd. (Shanghai, China). Bovine type II collagen, batch number: 2022); complete freund’s adjuvant, batch number: 7001; incomplete freund’s adjuvant, batch number: 7002 was purchased from Chondrex (Washington, USA). Paraformaldehyde, batch number: AR1069 was purchased from Wuhan Boshide Biological Technology Co., Ltd. (Wuhan, China). RPMI 1640 medium, batch number: C11965500BT; fetal bovine serum, batch number: 10270-106; penicillin-streptomycin, batch number: 15140122 was purchased from Thermo Fisher Scientific (Massachusetts, USA). 0.25% trypsin, batch number: 40126ES60 was purchased from Shanghai Yisheng Biotechnology Co., Ltd. (Shanghai, China). Lipopolysaccharide, batch number: L880 was purchased from Beijing Solarbio Science & Technology Co., Ltd. (Beijing, China). CCK-8 reagent kit, batch number: C0038 was purchased from Biyuntian Biotechnology Co., Ltd. (Shanghai, China). Mouse TNF-α ELISA Kit, batch number: 88-7324; Mouse IL-6 ELISA Kit, batch number: 88-7064 was purchased from Thermo Fisher Scientific (Massachusetts, USA). Mouse Sphk1 ELISA Kit was purchased from Jiangsu Enzyme-linked Immunosorbent Industrial Co., Ltd. (Yancheng, China). Mouse S1P ELISA Kit was purchased from Wuhan Feiyin Biotechnology Co., Ltd. (Wuhan, China). PF543, batch number: 567741 was purchased from Sigma-Aldrich (Shanghai) Trading Co., Ltd. (Shanghai, China).

### 4.2 Preparation of SIE

Following the preparation standard of “Snow Lotus Oral Liquid” and a previous report (Lee et al., 2011), the preparation process of SIE is as follows: The powder of dried SIH is passed through a 100-mesh sieve and then soaked in 10 times the amount of 65% ethanol for 30 min, heated to boiling point, and reflux extracted for 2 h. The hot solution is then filtered to obtain residue 1 and filtrate 1. The filtered residue 1 is then reflux extracted with 8 times the amount of 65% ethanol for another 2 h, and the hot solution is filtered to obtain residue 2 and filtrate 2. Finally, the filtered residue 2 is reflux extracted with 8 times the amount of 65% ethanol for an additional 2 h, and the hot solution is filtered to obtain filtrate 3. The three filtrates are combined, and the solvent is recovered under reduced pressure and concentrated until the ethanol odor is removed. The solution is then freeze-dried to obtain SIE lyophilized powder (yield 29.66%), which is dissolved in distilled water to the required concentration for use. To control the quality of SIE, a HPLC method was used (Supplementary Figure S1). Results showed that the mean content of chlorogenic acid per Gram of SIE was 8,118.2 μg, while that of rutin was 5,772.8 μg and that of luteolin was 1,451.9 μg (Supplementary Table S2).

### 4.3 Establishment of CIA mouse model and administration

Forty DBA/1J mice (approximately 6–8 weeks old, male), weighing 16–18 g, were purchased from Shanghai Slack Laboratory Animal Co., Ltd. (Shanghai, China). After a 7-day acclimation period, the mice were randomly divided into four groups: control, CIA, SIEL, and SIEH, with 10 mice in each group. According to the classic CIA modeling method (Brand et al., 2007), except for the control group, which received an equivalent stimulation of physiological saline, the other groups received an intradermal injection of 0.1 mL of a collagen emulsion composed of equal volumes of CII and Complete Freund’s Adjuvant (CFA) at a site 1.5 cm from the base of the tail, recorded as the first immunization. On the 21st day, a booster injection of 0.1 mL of a collagen emulsion composed of equal volumes of CII and Incomplete Freund’s Adjuvant (IFA) was administered at the distal end of the mouse tail, recorded as the second immunization. Administration of the treatment began on the same day as the first immunization. The control and CIA groups received physiological saline by gavage, while the SIE treatment groups received low (SIEL, 231 mg/kg) and high (SIEH, 2.31 g/kg) doses, based on the human recommended dose of 6 g/70 kg in the “Chinese Pharmacopoeia” (Committee, 2020), adjusted to 1x and 10x the human dose, respectively. The treatment was administered once daily for 42 consecutive days.

### 4.4 Measurement of body weight and evaluation of arthritis index

From day 21 to day 42, the body weight of the mice was measured using an electronic scale once a week. After the second immunization (day 21), the mice were scored once a week based on a mouse arthritis scoring standard for the ankle joint (Alabarse et al., 2018). The scoring grades range from 0 to 4 points: 0 points, no redness or swelling in the joints; 1 point, noticeable swelling and redness in one toe; 2 points, swelling in two toes or mild redness and swelling in the ankle joint; 3 points, moderate redness and swelling in the ankle joint, accompanied by toe swelling; 4 points, erythema and severe redness and swelling in the ankle joint and toes, with limb stiffness and inability to bear weight. The maximum cumulative arthritis score for the four paws of a mouse is 16 points. After the administration ended on day 42, the thickness of the mice’s four paws was measured using a vernier caliper. The results of the arthritis scores and paw thickness of the CIA mice were combined to verify the success of the model and the impact of SIE on CIA mice.

### 4.5 Histopathological examination

Ankle joint tissues were fixed in 4% paraformaldehyde, decalcified in 10% EDTA, embedded in paraffin, sectioned into 3 μm slices using a microtome, stained with hematoxylin and eosin (HE), and mounted with neutral resin. Images were collected using the Motic Digital Slide Assistant System, selecting appropriate fields of view. Observations included the clarity of the mouse ankle joint contours, the proliferation of synovial lining cells, the infiltration of inflammatory cells in the joint cavity, and the destruction of cartilage and bone.

### 4.6 Radiographic examination

Micro-computed tomography (Micro-CT, SKYSCAN 1272) was used to scan the hind paws of the mice to construct three-dimensional bone imaging and observe bone erosion.

### 4.7 Targeted sphingolipidomics analysis

#### 4.7.1 Preparation of plasma and spleen samples

A modified sample preparation method (Matyash et al., 2008) was carried out. 90 μL of mouse plasma (n = 5) was placed in a 10 mL glass test tube. 10 μL of mixed internal standards (IS), including SM(d18:1/12:0), Sph(d17:1)-1-P, Cer(d17:1/18:0), Cer(d18:1/8:0)-1-P, DhCer(d18:0/8:0) and HexCer (d18:1/17:0), were added to the sample to achieve final concentrations of 2000, 100, 10, 5, 10, and 50 ng/mL, respectively. The mixture was thoroughly combined, and 1.5 mL of methyl alcohol (MeOH) was added, followed by vortex mixing. 5 mL of methyl tert-butyl ether (MTBE) was then added, and the mixture was vortexed for 15 min and sonicated for 3 min 1.5 mL of ultrapure water was added to allow phase separation. The sample was centrifuged at 3,000 rpm for 5 min, and approximately 4 mL of the upper (organic) layer was collected. For the lower aqueous layer, a secondary extraction solution (MTBE: MeOH: H_2_O = 20:6:5, v/v/v) of 2 mL was added, vortexed for 5 min, and then centrifuged at 3,000 rpm for 5 min. The upper (organic) layers from both extractions were combined, vortexed thoroughly, dried under nitrogen at 37°C, and reconstituted with 100 µL of a MeOH: CHCl_3_ (1:1, v/v) solution. The sample was then centrifuged and analyzed.

For the spleen, the tissue (n = 5) was weighed, and three times the volume of physiological saline was added to create a tissue homogenate. 90 μL of the tissue homogenate was taken, 10 µL of mixed IS was added, mixed thoroughly, and the sample was processed using the same pretreatment method as for the plasma.

#### 4.7.2 HPLC-MS/MS detection conditions

After pretreatment, the biological samples were analyzed using an Agilent UPLC coupled with an Agilent 6460 triple quadrupole mass spectrometry system for relative quantification. The specific analytical conditions were as follows:

The chromatographic column used was a COPCELL CORE^®^PFP (2.1 mm × 30 mm, 2.7 μm), with the column temperature set at 35°C. The flow rate was 0.3 mL/min, and the injection volume was 5 µL. The mobile phase A consisted of 2 mM ammonium acetate buffer solution with 0.1% formic acid, while the mobile phase B was isopropanol/acetonitrile solution (2:5/v:v) containing 2 mM ammonium acetate and 0.1% formic acid. The gradient elution conditions for the mobile phase were as follows: 0–1 min (10% B to 10% B), 1–3 min (10% B to 90% B), 3–7 min (90% B to 98% B), 7–7.1 min (98% B to 10% B), and 7.1–10 min (10% B to 10% B).

The mass spectrometry scanning was conducted in dynamic multiple reaction monitoring (DMRM) mode with the following conditions: the ionization mode was electrospray ionization (ESI) and carried out in positive ion mode, capillary voltage at 4.0 kV, nebulizer gas (N_2_) flow rate at 10.0 L/min, desolvation gas (N_2_) flow rate at 30.0 L/min, desolvation temperature at 35°C, and the source temperature set at 35°C.

#### 4.7.3 Data acquisition and analysis

Data processing was conducted using Agilent Mass Hunter workstation software (version B.06.00). The identification of SPLs in mouse plasma and spleen was based on their accurate mass spectrometry and MS/MS data. Relative quantification in mass spectrometry mode was performed, and the relative content of 77 SPLs were estimated using Mass Hunter software based on the mass spectrometric peak areas of 77 SPLs and 6 IS from the HPLC-MS/MS data. Relative content calculated as follows: peak area of target SPLs/peak area of corresponding IS. Supplementary Table S3 lists the mass spectrometry parameters of the 77 SPLs and 6 IS compounds, including precursor ion, product ion, fragmentor, and collision energy. All relative quantification data were converted to Microsoft Excel format and imported into SIMCA 14.0 software (Umetrics, Umea, Sweden) for PCA and OPLS-DA analysis (Worley and Powers, 2012).

### 4.8 Cell culture and CCK8 assay

RAW 264.7 cells were purchased from the American Type Culture Collection (ATCC). The cells were cultured in 10 cm dishes using 1,640 complete medium at 37°C with 5% CO_2_, with daily passaging. For example, to prepare 100 mL of 1,640 complete medium, 1 mL penicillin-streptomycin, 10 mL fetal bovine serum (FBS), and 89 mL 1,640 medium were used to make a 10% 1,640 medium for cell culture. The medium was filtered through a 0.22 μm microporous membrane before use.

For the CCK8 assay, RAW 264.7 cells were seeded in 96-well plates at approximately 8 × 10^3^ cells and incubated overnight. Then, 1,640 medium containing SIE at concentrations of 0, 250, 500, 1,000, and 2000 μg/mL was added and incubated for 24 h. Afterward, 10 μL of CCK8 solution was added to the cells and incubated for 2 h, and absorbance was recorded at 450 nm. Cell viability was determined based on the optical density (OD) values.

### 4.9 The evaluation of protein levels of inflammation cytokines

RAW 264.7 cells were seeded in 12-well plates at approximately 3 × 10^4^ cells per well and cultured at 37°C with 5% CO_2_ for 24 h. Cells in the plates were pre-treated with or without LPS (1 μg/mL) for 24 h and then treated with various concentrations (0, 250, 500, 1,000 μg/mL) of SIE for another 24 h. The supernatants of the cell cultures from each group were collected and centrifuged at 1,200 rpm for 3 min. The supernatants were then collected, and the TNF-α and IL-6 protein levels were measured according to the instructions provided in the ELISA (Enzyme-linked immunosorbent assay) kits.

### 4.10 ELISA assay of SphK1 and S1P

RAW 264.7 cells were seeded in 12-well plates at approximately 3 × 10^4^ cells per well and cultured at 37°C with 5% CO_2_ for 24 h. Cells in the plates were pre-treated with or without LPS (1 μg/mL) for 24 h and then treated with various concentrations (0, 250, 500, 1,000 μg/mL) of SIE or PF543 (positive control, concentration 10 μM, SphK1 inhibitor) for another 24 h (Cheresh et al., 2020). The supernatants of the cell cultures from each group were collected and centrifuged at 1,200 rpm for 3 min. The supernatants were then collected, and the protein levels of SphK1 and the content of S1P were measured according to the instructions provided in the ELISA kits.

### 4.11 Statistical analysis

All statistical analyses were performed using SPSS software (SPSS Inc, Chicago, IL, USA). Data results were expressed as mean ± standard error of mean. Comparisons between groups were conducted using Dunnett’s t-test and the Mann-Whitney U test, with a significance level set at 0.05. A P-value <0.05 was considered statistically significant. Graphs were plotted using GraphPad Prism 8.0.2 software. Clustering heatmaps with symbols were generated using the Bioinformatics platform (https://www.bioinformatics.com.cn/).

## 5 Conclusion

In this study, we for the first time demonstrated that SIE could alleviate disease progression in CIA mice, and its regulatory effect on sphingolipid metabolism was involved in its anti-RA effects. More importantly, these findings provide pharmacological evidence for using SIH in the treatment of RA, and support the theory that targeting sphingolipid metabolism is a therapeutic strategy for RA.

## Data Availability

The original contributions presented in the study are included in the article/Supplementary Material, further inquiries can be directed to the corresponding author/s.
