# Supplementary material for: Investigating the underlying mechanisms of the ethanol extract of saussureae involucratae herba in anti-rheumatoid arthritis effect based on sphingolipidomics
Source: Front Pharmacol. 2025 Jun 18;16:1549437. doi: 10.3389/fphar.2025.1549437 (PMC12213489; doi:10.3389/fphar.2025.1549437)
Supplement: Supplementary file 1 [file DataSheet1.pdf]

Supplementary Material

1 Supplementary Figures and Tables

Table S1 High resolution mass data of components in S.I.

As our previously reported (Yang et al., 2022a) in the main article), UPLC-HRMS was used for identification.

No.	RT (min)	Formula	Experimental mass(m/z)	Theoretical mass(m/z)	Error (ppm)	MS^2	Component Name	Alcohol extract	Aqueous extract
H1	0.59	$C_7H_{11}O_6$	191.05522	191.05611	-4.67	192.05847(10),191.0551(100),173.04446(5),127.03866(4),111.00729(5),93.03305(2)	Quinic acid	+	+
H2	0.83	$C_{11}H_{11}O_4$	207.06564	207.06628	-3.10	208.06876(11),207.06548(100),179.03387(23),161.0233(11),135.04393(12),134.03586(9)	Isomer of methyl 4-(acetyloxymethyl)benzoate	+	+
H3*	0.96	$C_{16}H_{17}O_9$	353.08737	353.08781	-1.23	192.05856(8),191.05513(100),179.03424(2),161.02322(2),135.04378(1),85.02787(1)	Chlorogenic Acid	+	+
H4	0.96	$C_{16}H_{17}O_{10}$	369.08261	369.08272	-0.30	207.05025(15),195.02873(17),191.05513(100),173.04445(25),123.04372(23)	8-(glucosyloxy)-6-Methoxyumbelliferone	+	+
H5	1.36	$C_{20}H_{33}O_{11}$	449.20284	449.20288	0.10	269.1394(100),225.14896(25),209.11755(33),207.13828(15),89.02292(23),59.0123(44)		+	+

H6	1.37	$C_{25}H_{23}O_{12}$	515.11829	515.11950	-2.35	353.08765(15),341.08707(7),191.05513(86),179.03389(100),173.04445(19),135.04373(14)	1,3-Dicaffeoylquinic acid	+	+
H7	1.39	$C_{16}H_{17}O_{8}$	337.09317	337.09289	0.83	192.05847(8),191.05507(100),173.04445(10),163.03879(10),93.03303(18),59.01227(6)	5-p-Coumaroylquinic acid	+	+
H8	1.39	$C_{15}H_{21}O_{9}$	345.11896	345.11911	-0.42	166.05812(11),165.05449(100),148.04732(4),147.04367(21),144.04399(5),59.01239(5)	Isomer of di-*O*-Methylcrenatin	+	+
H9	1.54	$C_{16}H_{17}O_{9}$	353.08792	353.08781	0.35	191.05515(65),180.03711(7),179.03391(70),174.04799(8),173.04443(100),135.04378(32)	Isomer of chlorogenic Acid	+	+
H10	1.75	$C_{11}H_{15}O_{3}$	195.10208	195.10267	-3.01	196.09673(100),195.10176(40),195.04997(52),152.034(31),151.11153(66),108.04401(48)	Isomer of loliolide	+	+
H11	2.07	$C_{17}H_{19}O_{9}$	367.10358	367.10346	0.34	193.04959(9),192.05855(7),191.05515(100),173.04443(17),93.03306(24),87.00725(7)	3-*O*-Feruloylquinic acid	+	+
H12	2.12	$C_{10}H_{15}O_{7}$	247.08218	247.08233	-0.59	247.15498(97),205.00215(35),183.02885(39),129.05443(29),89.02293(40),87.04361(100)	Isomer of allyl methyl β-D-glucopyranosiduronate	+	+
H13	2.65	$C_{10}H_{19}O_{6}$	235.11844	235.11871	-1.16	217.06242(11),191.1071(14.69),174.09953(16),173.09608(100),73.02794(13),59.01227(13)	Butyl-β-D-fructopyranoside	+	+

H14	2.68	$C_{16}H_{17}O_8$	337.09305	337.09289	0.47	191.05495(5),174.0477(8),173.04448(100),163.03888(21),155.03383(5)	Isomer of 5-p-coumaroylquinic acid	+	+
H15	2.91	$C_{10}H_{15}O_7$	247.08215	247.08233	-0.71	185.08087(24),157.04942(34),139.03871(51),129.05437(61),111.00731(67),87.04361(100)	isomer of allyl methyl β-D-glucopyranosiduronate	+	+
H16	3.43	$C_{12}H_{21}O_6$	261.13436	261.13440	0.15	261.13434(6),244.06133(7),188.10008(7),187.09656(100),125.09576(58),109.01545(49)		+	+
H17*	3.44	$C_{17}H_{23}O_9$	371.13486	371.13476	0.28	163.03355(51),113.02302(30),101.02292(57),89.02288(100),71.01228(45),59.01231(92)	Syringin	+	+
H18	3.57	$C_9H_5O_4$	177.01850	177.01933	-4.42	178.02168(11),177.01825(100),149.02319(1),133.02808(7),105.03292(2)	Isomer of 5,7-dihydroxy-4H-chromen-4-one	+	+
H19	4.61	$C_{12}H_{21}O_6$	261.13436	261.13437	0.03	261.134(7),188.09993(11),187.09657(100),169.08592(6),126.09932(6),125.09576(61)		+	+
H20	5.03	$C_{10}H_7O_4$	191.03433	191.03498	-3.41	191.05513(100),191.03433(28),177.04182(4),147.04402(20),129.01799(3),111.00729(22)	Isomer of scopoletin	+	+
H21	5.05	$C_{15}H_{21}O_8$	329.12419	329.12424	0.15	329.09091(9),161.04446(4),113.02297(3),101.02292(6),85.02792(2),71.01226(4)		+	+

H22	5.10	$C_{19}H_{27}O_{11}$	431.15588	431.15601	0.29	210.08435(2),209.08112(23),194.05774(7),191.07106(2),176.04704(1),59.01231(100)		+	+
H23	5.10	$C_{25}H_{35}O_{12}$	527.21340	527.21344	0.08	113.02306(7),101.02292(28),89.02277(5),73.02795(22),71.01228(6),59.01232(100)		+	+
H24	5.35	$C_{25}H_{23}O_{12}$	515.11951	515.11950	0.02	353.08752(2),192.0585(4),191.05513(100),179.03397(5),161.02321(4),135.04395(1)	Isochlorogenic acid A	+	+
H25	5.40	$C_9H_5O_3$	161.02332	161.02442	-6.82	162.05479(4),162.0264(9),161.04477(3),161.02321(100),134.03212(3),133.02815(62)	Isomer of umbelliferone	+	+
H26	5.75	$C_{27}H_{25}O_7$	461.16058	461.16058	-2.77	119.03359(35),113.02293(22),101.02294(24)		+	+
H27	5.75	$C_{23}H_{33}O_{11}$	485.20313	485.20264	0.61	119.03359(35),113.02293(22),101.02294(24),89.02288(100),71.01229(35),59.01231(94)		+	+
H28	5.97	$C_{18}H_{25}O_{10}$	401.14532	401.14557	0.62	161.04436(35),113.02299(35),101.0229(54),89.02288(100),71.01228(62),59.0123(72)		+	+
H29	6.15	$C_{12}H_{13}O_6$	253.07181	253.07176	0.19	254.07471(8),253.0715(100),179.03398(30),161.02324(88),135.04384(62),134.0361(6)	Isomer of bis(2-hydroxyethyl) terephthalate	+	+
H30	6.59	$C_{33}H_{39}O_{21}$	771.19733	771.19893	-2.08	609.14502(50),463.0864(29),462.08096(100),301.035(89),300.02686(44),299.01962(44)	Quercetin3-rutinoside-7-glucoside	+	+

H31	6.60	$C_{18}H_{25}O_{10}$	401.14532	401.14554	0.55	269.10336(56),161.04431(93),113.02303(36),101.02293(100),71.01234(75),59.0124(41)		+	+
H32	6.68	$C_{13}H_{25}O_{8}$	309.15549	309.15573	0.77	249.13434(2),161.04475(1),101.0229(3),71.01233(2),60.01562(1),59.01229(100)		+	+
H33	7.11	$C_8H_7O_4$	167.03415	167.03498	-4.98	NF	Vanillic acid	+	+
H34	7.19	$C_{21}H_{29}O_{8}$	409.18695	409.18679	0.39	248.13657(17),247.13367(100),203.1432(16),101.02282(10),71.01234(16),59.01231(28)	11β,13-Dihydrodehydrocostuslactone-8-*O*-β-D-glucoside	+	+
H35	7.30	$C_9H_5O_3$	161.02339	161.02442	-6.38	162.02661(10),161.02321(100),133.02811(5),85.0281(3),73.02795(2)	Isomer of umbelliferone	+	+
H36	7.30	$C_{25}H_{25}O_{6}$	421.16566	421.16391	-4.16	197.04483(44),179.03436(7),163.40208(5),161.02318(100),61.98678(69),59.0124(10)		+	+
H37	7.30	$C_{21}H_{33}O_{10}$	445.20792	445.20810	0.40	153.09088(100),152.08305(35),101.02296(28),89.02293(28),71.01228(31),59.01231(79)		+	+
H38	7.33	$C_{18}H_{15}O_{8}$	359.07745	359.07724	0.58	359.20761(11),197.04456(33),179.03386(16),161.02321(100),72.99152(11),59.01231(9)	Rosmarinic acid	+	+
H39	7.42	$C_{20}H_{25}O_{6}$	361.16629	361.16566	1.74	362.16754(15),361.22537(29),361.16748(100),162.02658(16),161.02301(20)	Isomer of secoisolariciresinol	+	+

H40	7.64	$C_{27}H_{25}O_7$	461.16058	461.16058	-4.42	119.03356(36),113.02296(24),101.02293(27)		+	+
H41	7.66	$C_{23}H_{33}O_{11}$	485.20284	485.20264	-0.40	119.03356(36),113.02296(24),101.02293(27),89.02288(100),71.01228(36),59.01231(97)		+	+
H42	7.77	$C_{23}H_{33}O_9$	453.21301	453.21304	0.08	453.21335(17),412.20477(16),411.20221(100),393.19193(49),249.14922(54),59.01231(29)		+	+
H43	7.77	$C_{17}H_{23}O_6$	323.15018	323.15027	0.52	NF		+	+
H44	7.78	$C_{24}H_{33}O_{11}$	497.20325	497.20325	0.84	453.21298(38),412.20587(15),411.20236(100),393.19235(60),249.14906(39)	Iridodial glucoside tetraacetate	+	+
H45	7.84	$C_9H_5O_4$	177.01843	177.01933	-5.10	180.0374(12),179.03386(33),178.02156(11),177.01828(100),135.04391(21),93.03297(11)	5,7-Dihydroxy-4H-chromen-4-one	+	+
H46	7.88	$C_{22}H_{25}O_8$	417.15387	417.15549	-3.89	381.1777(69),249.13368(100),187.09634(67),161.04474(13),101.02296(34)	Syringaresinol	+	+
H47*	8.01	$C_9H_5O_3$	161.02330	161.02442	-6.94	162.02646(9),161.02321(100),134.03149(3),133.02814(63),73.02786(2)	Umbelliferone	+	+
H48*	8.01	$C_{25}H_{23}O_{12}$	515.11945	515.11950	-0.10	354.09088(9),353.08759(39),192.0585(9),191.05511(100),179.03389(64),135.04376(11)	Isochlorogenic acid C	+	+

H49	8.43	$C_{23}H_{21}O_{13}$	505.09949	505.09877	1.44	461.11221(13),316.05383(8),315.05075(100),314.04388(48),151.00247(30)	Jaceosidin-6’-methyl-7-*O*-glucuronide	+	+
H50	8.44	$C_{17}H_{23}O_{6}$	323.15027	323.15027	0.80	NF		+	+
H51	8.56	$C_{15}H_{19}O_{3}$	247.13397	247.13397	0.01	248.13699(19),247.13364(100),204.14674(16),203.14331(95),201.12738(3),187.11214(4)	Involucratolactone	+	+
H52	8.89	$C_{21}H_{31}O_{8}$	411.20248	411.20248	0.10	412.11673(14),411.11484(100),351.0936(85),249.07138(13),219.05083(14),147.02832(13)	lemmonin C	+	+
H53	8.92	$C_{22}H_{19}O_{13}$	491.08365	491.08311	1.09	491.08310(48),345.02380(67),344.01736(83),300.02768(100),301.03415(49)	Quercetin 3-*O*-glucuronide 6"-methyl ester	+	+
H54*	8.94	$C_{25}H_{23}O_{12}$	515.11945	515.11950	-0.10	353.0878(25),191.05521(24),179.03394(73),173.04448(100),155.03398(5),135.04385(12)	1,5-Dicaffeoylqunic acid	+	+
H55	9.22	$C_{21}H_{17}O_{13}$	477.06787	477.06747	0.85	302.03836(12),301.03516(100),178.99763(10),151.00252(11)	Quercetin-3-*O*-glucuronide	+	+
H56	9.42	$C_{10}H_{7}O_{3}$	175.03941	175.03897	-3.76	NF	4-Methylumbelliferone	+	+
H57	9.49	$C_{25}H_{23}O_{12}$	515.11963	515.11950	0.25	353.08804(18),191.05516(40),179.03394(85),173.04448(100),161.02319(15),135.04376(15)	Isochlorogenic acid B	+	+

H58	9.52	$C_{22}H_{19}O_{13}$	491.08377	491.08311	1.34	491.15982(25),302.03867(13),301.03494(90),300.02725(100)	Isomer of quercetin 3-*O*-glucuronide 6"-methyl ester	+	+
H59	9.69	$C_{17}H_{19}O_{9}$	367.10388	367.10346	1.16	368.10724(9),367.10327(58),180.03725(8),179.03397(100),161.02321(13),135.04382(37)	1-*O*-Methyl chlorogenic acid	+	+
H60	9.72	$C_{23}H_{21}O_{13}$	505.09946	505.09877	1.38	506.26929(27),505.26471(48),119.03324(39),101.02287(33),89.02287(62),59.01231(100)	Isomer of jaceosidin-6'-methyl-7-*O*-glucuronid	+	+
H61	9.72	$C_{24}H_{21}O_{15}$	549.08948	549.08859	1.62	508.59933(2),302.03967(7),301.03381(65),300.02744(100),151.00252(3)	Quercetin 3-*O*-(6-*O*-malonyl-β-D-glucoside)	+	+
H62	9.75	$C_{11}H_{15}O_{3}$	195.10208	195.10267	-3.01	195.10126(100),180.04237(19),165.0547(38),151.11151(51),133.10071(82)	Loliolide	+	+
H63	9.81	$C_{33}H_{39}O_{21}$	771.20013	771.19893	1.55	670.42194(7),488.01974(6),436.46506(7),301.03476(100),300.02728(78)	Isomer of quercetin-3-rutinoside-7-glucoside	+	+
H64	10.03	$C_{17}H_{11}O_{4}N_{2}$	307.07275	307.07243	1.04	306.17105(10),264.08533(12),263.08258(100),234.07423(10),233.07149(96),59.01228(12)	Flazin	+	+
H65	10.18	$C_{25}H_{23}O_{12}$	515.11932	515.11950	-0.35	353.08749(21),255.0659(22),203.03416(36),191.05513(24),179.03387(75),173.04446(100)	1,4-Dicaffeoylqunic acid	+	+

H66*	10.23	$C_{27}H_{29}O_{16}$	609.14594	609.14611	-0.28	609.14594(18),302.03915(3),301.03482(59),300.02734(100),178.99792(4),151.00252(4)	Rutin	+	+
H67	10.28	$C_{28}H_{31}O_{17}$	639.15808	639.15667	2.20	NF	Isorhamnetin-3,4'-diglucoside	+	+
H68	10.38	$C_{15}H_{11}O_{6}$	287.05609	287.05611	-0.07	287.05624(19),260.06458(14),259.0611(88),243.06635(17),125.02302(100),59.01231(24)	Isomer of Eriodictyol	+	+
H69*	10.38	$C_{21}H_{19}O_{12}$	463.08853	463.08820	0.71	463.08881(7),302.03891(6),301.03445(57),300.02737(100),178.99739(3),151.00244(4)	Isoquercitrin	+	+
H70	10.41	$C_{15}H_{21}O_{3}$	249.14929	249.14962	-1.32	250.15309(19),249.14915(100),233.94821(11),203.14287(8),187.14841(8),73.02803(10)		+	+
H71	10.57	$C_{15}H_{19}O_{3}$	247.13388	247.13397	-0.36	248.13658(4),247.13362(27),204.14671(10),203.14334(100),163.11136(5),149.09651(3)	Xuelianlactone	+	+
H72	10.67	$C_{20}H_{23}O_{7}$	375.14532	375.14493	1.05	328.12692(17),327.12366(100),312.10028(45),195.06535(28),165.0545(100),164.04663(18)	Isomer of berchemol	+	+
H73	10.83	$C_{17}H_{23}O_{6}$	323.15027	323.15027	0.80	NF		+	+
H74	11.04	$C_{14}H_{19}O_{3}$	235.13383	235.13397	-0.59	236.13664(11),235.13342(100),192.14645(3),191.14317(21),175.11151(4),57.03302(10)	Guaianolide	+	+

H75	11.16	$C_{20}H_{23}O_7$	375.14542	375.14493	1.32	327.12390(3),195.06531(16),180.04184(6),179.07047(7),166.05791(7),165.05457(100),150.03091(24)	Isomer of berchemol	+	+
H76	11.19	$C_{10}H_7O_3$	175.03931	175.03897	3.34	NF	Isomer of 4-methylumbelliferone	+	+
H77	11.39	$C_{21}H_{21}O_7$	385.12881	385.12928	-1.21	385.13223(56),297.11655(100),233.11771(39),135.98219(50),87.00724(47),74.98943(38)	Edultin	+	+
H78	11.42	$C_{28}H_{35}O_{13}$	579.20905	579.20831	1.27	NF	Isomer of syringaresinol 4-*O*-β-D-glucopyranoside	+	+
H79	11.66	$C_{22}H_{41}O_{10}$	465.27052	465.27060	0.17	466.27542(25),465.26993(84),447.2598(25),101.02306(32),71.01221(16),59.01232(100)		+	+
H80	11.76	$C_{10}H_7O_4$	191.03429	191.03498	-3.66	192.0372(12),191.05484(36),191.03409(100),176.00984(8),135.85411(4),111.00729(7)	Scopoletin	+	+
H81	11.79	$C_{27}H_{29}O_{13}$	561.16180	561.16180	0.78	369.09778(19),191.05516(100),173.04449(16),161.02328(12),160.01549(10)		+	+
H82	11.83	$C_{20}H_{23}O_6$	359.14990	359.15001	-0.31	359.14999(42),344.12613(100),313.10794(15),189.05447(14),159.04391(14)	Lariciresinol	+	+
H83	11.97	$C_{26}H_{27}O_{12}$	531.15080	531.15094	0.27	339.08713(13),191.05521(100),177.01779(9),173.04468(14),160.01566(9),93.03309(13)		+	+

H84	12.05	$C_{27}H_{29}O_{15}$	593.15173	593.15119	0.91	594.15356(3),593.15179(10),286.04221(17),285.03888(100),284.03159(54),151.00240(2)	Lonicerin	+	+
H85	12.10	$C_{19}H_{21}O_{6}$	345.13458	345.13436	0.63	345.22595(7),192.07306(1),191.06963(100),176.04613(15),123.04377(4)	Cynaropicrin	+	+
H86	12.10	$C_{20}H_{23}O_{7}$	375.14523	375.14493	0.81	315.12305(3),,217.00247(5),192.0739(9),191.07034(100),179.07019(4),176.04665(14),123.04369(15)	Berchemol	+	+
H87*	12.22	$C_{21}H_{19}O_{11}$	447.09344	447.09328	0.35	447.09457(6),302.03845(14),301.03445(94),300.02734(100),178.9973(3),151.00209(5)	Quercitrin	+	+
H88	12.55	$C_{28}H_{31}O_{16}$	623.16223	623.16176	0.76	623.16345(4),316.05441(9),315.05096(100),314.04315(40),300.02658(6),299.02032(4)	Isorhamnetin-3-*O*-rutinoside	+	+
H89	12.74	$C_{21}H_{21}O_{11}$	449.10944	449.10893	1.13	288.05984(15),287.05618(76),151.0024(100),135.04378(22)	Eriodictyol-7-glucoside	+	+
H90	12.78	$C_{22}H_{21}O_{12}$	477.10434	477.10385	1.03	478.10788(6),477.10416(38),315.04941(27),314.04318(100),299.01974(5),285.04034(6)	Nepetin-7-*O*-glucoside	+	+
H91	13.56	$C_{21}H_{19}O_{10}$	431.09879	431.09837	0.97	432.10156(18),431.22818(20),431.09763(100),269.04486(53),268.03754(81)	Apigenin-7-*O*-β-D-glucoside	+	+
H92	13.70	$C_{11}H_{11}O_{3}$	191.07065	191.07137	-3.76	NF	Isomer of 2,6-dimethoxy-4-[(1E)-1-propen-1-yl]phenol	+	+

H93	13.86	$C_{21}H_{19}O_{11}$	447.09372	447.09328	0.97	447.09317(3),301.03479(12),300.02765(14),286.04395(18),285.04037(100),59.0123(9)	Isomer of quercitrin	+	+
H94	13.91	$C_{28}H_{35}O_{13}$	579.20880	579.20831	0.84	418.15936(9),417.15549(38),402.13184(16),182.05301(10),181.04959(100),166.02596(9)	Syringaresinol 4-*O*-β-D-glucopyranoside	+	+
H95	14.50	$C_{21}H_{19}O_{10}$	431.09879	431.09837	0.97	431.09695(9),286.04428(11),285.04019(100),284.0325(66),255.02911(5)	Isomer of apigenin-7-*O*-β-D-glucoside	+	+
H96	14.64	$C_{20}H_{25}O_{6}$	361.16599	361.16566	0.91	362.16898(16),361.16568(100),346.14206(41),165.05458(57),132.0289(20),122.03604(22)	Secoisolariciresinol	+	+
H97	14.67	$C_{22}H_{21}O_{11}$	461.10941	461.10893	1.03	461.1088(79),299.05551(27),298.04797(31),297.0405(19),284.03104(23),283.02475(100)	Hispidulin-7-*O*-glucoside	+	+
H98	14.91	$C_{15}H_{11}O_{6}$	287.05640	287.05611	1.01	152.00568(7),151.0024(100),136.04706(5),135.04381(64),125.02289(4),107.01235(7)	Eriodictyol	+	+
H99	15.01	$C_{15}H_{21}O_{3}$	249.14941	249.14962	-0.83	250.15263(12),249.14922(100),231.13887(5),205.15944(3),203.14336(7),187.14809(10)		+	+
H100	15.27	$C_{20}H_{23}O_{6}$	359.15005	359.15001	0.11	330.14246(26),329.13925(98),192.0782(52),178.06248(65),175.07535(100),160.05173(44)	Isomer of lariciresinol	+	+
H102	15.34	$C_{25}H_{35}O_{11}$	511.21820	511.21820	-0.56	NF		+	+

H101	15.34	$C_{23}H_{23}O_{12}$	491.11963	491.11950	0.27	491.11899(74),476.09604(46),328.05933(34),327.05264(23),314.04257(24),313.03525(100)	Jaceosidin-7-*O*-glucoside	+	+
H103	15.38	$C_{26}H_{31}O_{11}$	519.18652	519.18718	-1.28	358.13763(23),357.13431(100),342.11093(7),221.08124(9),137.05948(7)	Pinoresinol-β-D-glucoside	+	+
H104	16.25	$C_{20}H_{21}O_{6}$	357.13464	357.13436	0.78	221.08118(59),206.05797(22),203.07047(100),192.0779(27),191.07043(31),123.04374(33)	Isomer of matairesinol	+	+
H105	16.26	$C_{20}H_{23}O_{6}$	359.15079	359.15001	2.17	NF	Isomer of lariciresinol	+	+
H106	16.34	$C_{20}H_{25}O_{6}$	361.16577	361.16566	0.30	NF	Isomer of secoisolariciresinol	+	+
H107	16.60	$C_{16}H_{11}O_{7}$	315.05118	315.05103	0.49	316.05453(4),315.05096(19),301.03055(20),300.02731(100)	Nepetin	+	+
H108*	16.78	$C_{15}H_{9}O_{6}$	285.04059	285.04046	0.45	286.04379(19),285.04037(100),199.03938(1),175.03944(1),151.00233(2),133.02773(3)	Luteolin	+	+
H109*	16.88	$C_{15}H_{9}O_{7}$	301.03540	301.03538	0.08	302.03851(15),301.03516(90),178.99748(57),152.00575(9),151.00238(100),121.02809(18)	Quercetin	+	+
H110	17.33	$C_{15}H_{19}O_{3}$	247.13393	247.13397	-0.15	248.13721(6),247.1337(43),204.14651(15),203.14334(100),149.09599(14),137.09569(8)	8α-Hydroxy-11βH-11,13-dihydrodehydrocostuslactone	+	+

H111	17.67	$C_{11}H_{11}O_4$	207.06557	207.06628	-3.44	208.06876(12),207.06548(100),179.03387(23),161.0233(11),135.04393(12),134.03586(9)	Methyl 4-(acetyloxymethyl)benzoate	+	+
H112	18.26	$C_{23}H_{33}O_{10}$	469.20779	469.20792	-0.28	247.13403(6),113.02299(7),101.02291(9),89.02289(6),71.0123(8),59.0123(100)		+	+
H113	18.38	$C_{16}H_{31}O_4$	287.22278	287.22278	-0.01	288.22617(16),287.22266(100),287.05331(1),286.2106(3),181.15897(4),169.15846(3)		+	+
H114	18.49	$C_{15}H_{11}O_5$	271.06134	271.06120	0.53	271.19153(100),271.06137(81),253.18098(56),209.19025(33),151.00224(58),119.04882(22)	Naringenin	+	+
H115	18.75	$C_{22}H_{25}O_8$	417.15546	417.15549	-0.07	402.13177(12),387.10861(22),182.05273(6),181.04962(100),166.02599(36),152.04709(5)	Syringaresinol	+	+
H116	18.75	$C_{27}H_{33}O_{11}$	533.20300	533.20283	0.31	372.15219(8),371.14932(61),356.12564(18),235.09702(9),136.05164(13),83.01227(100)	Arctiin	+	+
H117	18.75	$C_{29}H_{37}O_{13}$	593.22418	593.22396	0.36	372.15274(12),371.14954(79),356.12665(10),235.09709(9),83.01227(46),59.01229(100)		+	+
H118	18.92	$C_{20}H_{21}O_6$	357.13400	357.13436	-1.03	357.13431(1),342.11023(6),175.07515(1),152.04225(6),151.0388(100),136.01526(20)	Isomer of matairesinol	+	+
H120	19.01	$C_{23}H_{33}O_{10}$	469.20764	469.20792	-0.60	119.03356(28),113.02292(21),101.02296(22),89.02284(86),71.01227(31),59.01229(100)		+	+

H119	19.01	$C_{21}H_{29}O_8$	409.18631	409.18679	-1.18	119.03352(27),113.02296(23),101.0229(27),89.02286(100),71.01227(44),59.0123(80)	Isomer of 11β,13-dihydrodehydrocostuslactone-8-*O*-β-D-glucoside	+	+
H121*	19.81	$C_{16}H_{11}O_6$	299.05609	299.05611	-0.07	300.05923(2),299.05585(14),285.03604(15),284.03253(100),212.04758(1),136.98683(2)	Hispidulin	+	+
H122*	20.04	$C_{15}H_9O_5$	269.04575	269.04555	0.76	270.04865(7),269.04544(100),225.05475(2),151.00235(2),149.02315(2),117.03282(2)	Apigenin	+	+
H123	20.05	$C_{20}H_{26}NO_4$	344.18680	344.18673	0.20	345.22653(1),254.18428(1),197.72614(1),115.05803(5),114.05458(100)	Involucratine	+	+
H124	20.73	$C_{17}H_{13}O_7$	329.06686	329.06668	0.56	329.2327(4),329.06659(12),315.04669(13),314.04315(100),299.0195(15),271.02521(2),	Jaceosidin	+	+
H125	20.78	$C_{15}H_9O_6$	285.04077	285.04046	1.08	286.04382(11),285.20789(2),285.04037(100),284.26663(1),257.04669(1),151.00209(3)	Kaempferol	+	+
H126	21.00	$C_{16}H_{11}O_6$	299.05630	299.05611	0.63	300.05966(7),299.22318(7),299.05603(62),285.03561(8),284.0325(100),256.03741(2)	Isomer of hispidulin	+	+
H127	21.29	$C_{20}H_{21}O_6$	357.13437	357.13436	0.02	357.13425(32),342.11029(16),221.08138(14),137.05946(32),122.03596(11),83.01227(100)	Matairesinol	+	+

H128	21.35	$C_{18}H_{31}O_8$	375.20244	375.20251	0.18	217.00262(2),161.04482(1),113.02309(1),101.02293(3),71.01224(3),59.01229(100)		+	+
H129	21.61	$C_{17}H_{27}O_5$	311.18640	311.18665	0.81	311.18625(11),294.17926(5),293.1759(34),268.19992(12),267.19644(100),249.18559(4)		+	+
H130	21.74	$C_{16}H_{11}O_7$	315.05127	315.05103	0.78	316.05414(14),315.05106(73),301.03073(19),300.0274(100),151.00247(3)	Isomer of nepetin	+	+
H131	21.77	$C_{18}H_{25}O_4$	305.17583	305.17593	0.32	305.17578(14),250.15282(10),249.14929(69),135.0802(100),125.09581(21),97.0644(17)		+	+
H132	22.22	$C_{11}H_{11}O_3$	191.07066	191.07137	-3.70	192.07402(11),191.0704(100),163.03886(24),145.02811(23),119.04893(16),118.04111(10)	2,6-Dimethoxy-4-[(1E)-1-propen-1-yl]phenol	+	+
H133	22.66	$C_{21}H_{44}O_{17}N$	582.26147	582.26093	-0.93	462.20367(31),343.1488(18),342.14569(91),316.16638(17),145.02814(39),119.04881(100)		+	+
H134	22.67	$C_{15}H_{19}O_2$	231.13872	231.13905	-1.44	NF	Isomer of dihydrodehydrocostuslactone	+	+
H135	23.32	$C_{17}H_{23}O_5$	307.15518	307.15510	0.27	NF		+	+
H136	24.14	$C_{20}H_{23}O_6$	359.15179	359.15001	4.95	NF	Isomer of lariciresinol	+	+

H137	24.44	$C_{15}H_{19}O_2$	231.13884	231.13905	-0.92	NF	Isomer of dihydrodehydrocostuslactone	+	+
H138*	25.20	$C_{21}H_{23}O_6$	371.14948	371.15001	-1.43	371.14935(5),356.1264(9),151.07512(17),136.05164(35),121.02805(6),83.01228(100)	Arctigenin	+	+
H139	25.83	$C_{23}H_{24}O_4N_2$	392.17459	392.17459	1.11	NF		+	+
H140	26.18	$C_{17}H_{13}O_6$	313.07202	313.07176	0.83	313.23843(60),313.07184(75),299.05179(23),298.04803(100),283.02475(46),183.13814(23)	5,7-Dihydroxy-6,4'-dimethoxyflavone	+	+
H141	26.33	$C_{25}H_{35}O_{11}$	511.21811	511.21820	-0.73	113.02299(4),101.0229(19)		+	+
H142	26.42	$C_{15}H_{19}O_2$	231.13867	231.13905	-1.66	233.15361(11),232.14098(35),232.04208(26),231.13754(100),59.01202(22),	Dihydrodehydrocostuslactone	+	+
H143	27.30	$C_{15}H_{21}O_2$	233.15422	233.15470	-2.07	234.15764(10),233.15411(100),	Costic acid	+	+
H144	27.54	$C_{18}H_{33}O_4$	313.23862	313.23843	0.60	314.24173(19),313.23846(85),295.22772(12),202.11578(13),201.1124(100),171.10158(11)		+	+
H145	27.68	$C_{15}H_{19}O_2$	231.13907	231.13905	0.07	NF	Isomer of dihydrodehydrocostuslactone	+	+

H146	28.04	$C_{18}H_{29}O_3$	293.21234	293.21222	0.42	294.21518(12),293.21234(41),276.20493(23),275.20132(95),236.1732(17),235.16982(100)		+	+
H147	28.10	$C_{15}H_9O_5$	269.04584	269.04555	1.09	270.21573(19),269.2121(100),268.14594(4),225.22148(9),223.20616(13)	Isomer of Apigenin	+	+
H148	28.21	$C_{16}H_{13}O_4$	269.08224	269.08193	1.14	270.21573(19),269.2121(100),268.14594(4),225.22148(9),223.20616(13)	Imperatorin	+	+
H149	28.42	$C_{18}H_{31}O_3$	295.22772	295.22787	-0.50	296.23132(21),295.22769(91),294.21573(18),278.22073(22),277.21716(100),171.10161(60)		+	+
H150	28.51	$C_{18}H_{29}O_3$	293.21246	293.21222	0.83	294.21536(27),293.21219(100),113.0957(27),249.22208(7),195.13855(5),167.1068(6)		+	+
H151	29.14	$C_{15}H_{21}O_2$	233.15431	233.15470	-1.69	NF	Isomer of costic acid	+	+

RT, retention time; “*”, confirmed by authentic standard compound; “NF”, not found.

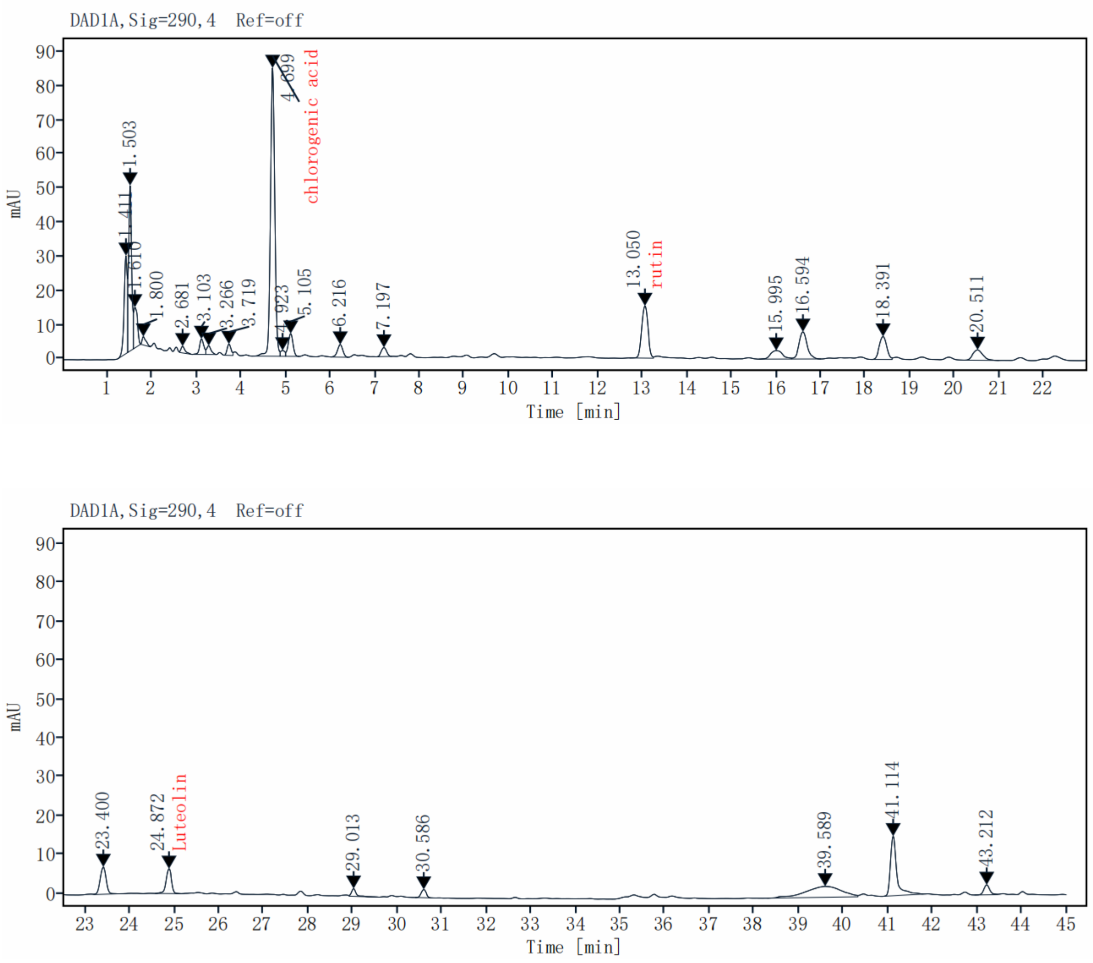

Figure S1. An HPLC method developed for quality control of SIE. HPLC chromatograms of SIE. HPLC analysis was performed on an Agilent 1200 system coupled with a C18 column (4.6× 150 mm2, 4 μm; Agilent Poroshell 120 EC-C18) maintained at 35℃. Elution was performed with a mobile phase of A (0.1% (v/v) formic acid aqueous solution) and B (acetonitrile) under a gradient program of 90% A at 0-20 min, 78% A at 20-32 min, followed by 51% A at 32-45 min. The flow rate was 1.0 mL/min, and the injection volume was 10 μL. Diode-array detector (DAD) was used to identify compounds containing conjugated double bonds. DAD spectra were measured over the wavelength range of 290 nm. The HPLC chromatogram showed that chlorogenic acid, rutin and luteolin were present in SIE.

Table S2. The content of three active components in SIE

ID	Compound name	Retention time (min)	Peak area	Response factor	Concentration (μg/mL)	Content (μg/g)
1	Chlorogenic acid	4.669	587.4956	36.18394	16.236	8118.2
2	Rutin	13.050	149.1806	12.92099	11.546	5772.8
3	Luteolin	24.872	52.2019	18.62544	2.904	1451.9

Table S3. ESI-MS MS/MS Dynamic MRM conditions of 77 sphingolipids (SPLs) and 6 internal standard (IS) compounds.

ID	Compound name	Precursor Ion	Product Ion	Fragmento r	CE
1	SM (d18:1/2:0)	507.3	183.9	200	33
2	SM (d18:1/4:0)	535.6	183.9	160	30
3	SM (d18:1/14:0)	675.6	183.9	160	30
4	SM (18:1/15:0)	689.6	184.1	160	30
5	SM (d18:1/14:1)9Z	690	183.9	160	30
6	SM (18:1/16:1)	701.6	184.1	160	30
7	SM (d18:1/16:0)	703.5	183.9	190	33
8	SM (d18:1/17:0)	717.5	183.9	100	30
9	SM (18:1/18:2)	727.6	184.1	160	30
10	SM (d18:1/18:1)11Z	730.1	183.9	160	30
11	SM (d18:1/18:0)	731.6	183.9	180	33
12	SM (18:1/19:1)	743.5	184.1	160	30
13	SM (18:1/19:0)	745.6	184.1	160	30

14	SM (18:1/20:1)	757.6	184.1	160	30
15	SM (d18:1/20:0)	759.6	183.9	160	30
16	SM (18:1/21:0)	773.6	184.1	160	30
17	SM (18:1/22:2)	783.6	184.1	160	30
18	SM (18:1/22:1)	785.7	184.1	160	30
19	SM (18:1/22:0)	789.7	184.1	160	30
20	SM (18:1/23:1)	799.7	184.1	160	30
21	SM (18:1/23:0)	801.6	183.9	160	30
22	SM (d18:1/24:2) 5Z9Z	812.2	183.9	100	24
23	SM (d18:1/24:1)	813.6	183.9	100	24
24	SM (d18:1/24:0)	815.6	183.9	160	24
25	SM (d18:1/26:1)	841.6	183.9	200	35
26	SM (d18:1/26:0)	843.6	183.9	200	35
27	Sph (d17:1)	286	267.8	100	7
28	DhSph (d17:0)	287.9	270	110	11
29	DhSph (d18:0)	302.1	284.2	120	10
30	S1P (d18:1)	380.3	264.1	110	12
31	Cer (d18:1/2:0)	342.1	324.1	100	2
32	Cer (d18:1/4:0)	370.2	352.1	100	2
33	Cer (d18:1/10:0)	168.1	112.1	130	24
34	Cer (d18:1/14:0)	510.4	492.1	120	6

35	Cer (d18:1/16:0)	538.4	520.1	140	6
36	Cer (d18:1/18:1)	564.4	546.2	130	6
37	Cer (d18:1/18:0)	566.4	548.3	150	12
38	Cer (d18:1/20:0)	594.7	576.7	140	8
39	Cer (d18:1/22:0)	622.5	604.2	140	8
40	Cer (d17:1/24:1)	634.5	616.6	140	10
41	Cer (d18:2/24:1)	646.6	262.3	140	8
42	Cer (d18:1/24:1)	648.7	630.2	140	12
43	Cer (d18:2/24:0)	648.6	262.3	140	8
44	Cer (d18:1/24:0)	650.7	632.3	140	8
45	Cer (d18:1/26:0)	678.8	660.3	140	8
46	Cer (d18:1/16:0)-1-P	618.1	264.1	110	31
47	Cer (d18:1/22:0) -1-P	702.1	264.1	140	30
48	Cer (d18:1/24:1)-1-P	728.1	264.1	140	30
49	Cer (d18:1/24:0)-1-P	730.1	264.1	140	30
50	Cer (d18:1/26:1)-1-P	756.1	264.1	140	30
51	DhCer (d18:0/4:0)	372.2	354.2	140	10
52	DhCer (d18:0/6:0)	400.1	382.2	130	10
53	DhCer (d18:0/12:0)	484.2	466.2	140	15
54	DhCer (d18:0/14:0)	512.2	494.2	140	15
55	DhCer(d18:0/16:0)	540.2	522.4	160	18

56	DhCer (d18:0/18:1)	566.2	548.4	140	18
57	DhCer (d18:0/18:0)	568.2	550.3	140	18
58	DhCer (d18:0/20:0)	596.2	578.2	140	20
59	DhCer (d18:0/22:0)	624.2	606.2	140	20
60	DhCer (d18:0/24:1)	650.3	632.5	150	24
61	DhCer (d18:0/26:0)	680.2	662.2	140	25
62	HexCer (d18:1/18:0)	728.2	264.1	150	39
63	HexCer (d18:1/20:0)	756.2	264.1	100	36
64	HexCer (d18:1/22:0)	784.2	264.1	100	36
65	HexCer (d18:1/24:1)	810.2	264.1	100	36
66	HexCer (d18:2/24:0)	810	262.3	130	40
67	HexCer (d18:0/24:1)	812	264.4	100	36
68	Hex_2Cer (d18:1/16:0)	863	264.4	100	36
69	Hex_2Cer (d18:1/18:0)	891	264.4	100	36
70	Hex_2Cer (d18:0/20:0)	921	266.4	100	36
71	Hex_2Cer (d18:0/22:0)	949	266.4	100	36
72	Hex_2Cer (d18:1/ 22:0)	947	264.4	100	36
73	Hex_2Cer (d18:1/20:1)	917	264.4	100	36
74	Hex_2Cer (d18:1/20:0)	919	264.4	100	36

75	Hex_2Cer (d18:1/22:1)	945	264.4	100	36
76	Hex_2Cer (d18:1/24:2)	971	264.4	100	36
77	Hex_2Cer (d18:1/24:1)	973	264.4	100	36
IS1	SM (d18:1/12:0)	647.5	184	100	20
IS2	Cer (17:1/18:0)	552.4	250.3	120	20
IS3	Cer (d18:1/8:0)-1-P	506	264.3	90	35
IS4	Sph (d17:1)-1-P	365.9	250	100	15
IS5	DhCer (d18:1/8:0)	428.5	410.6	110	15
IS6	HexCer (d18:1/17:0)	715.5	264.4	120	45

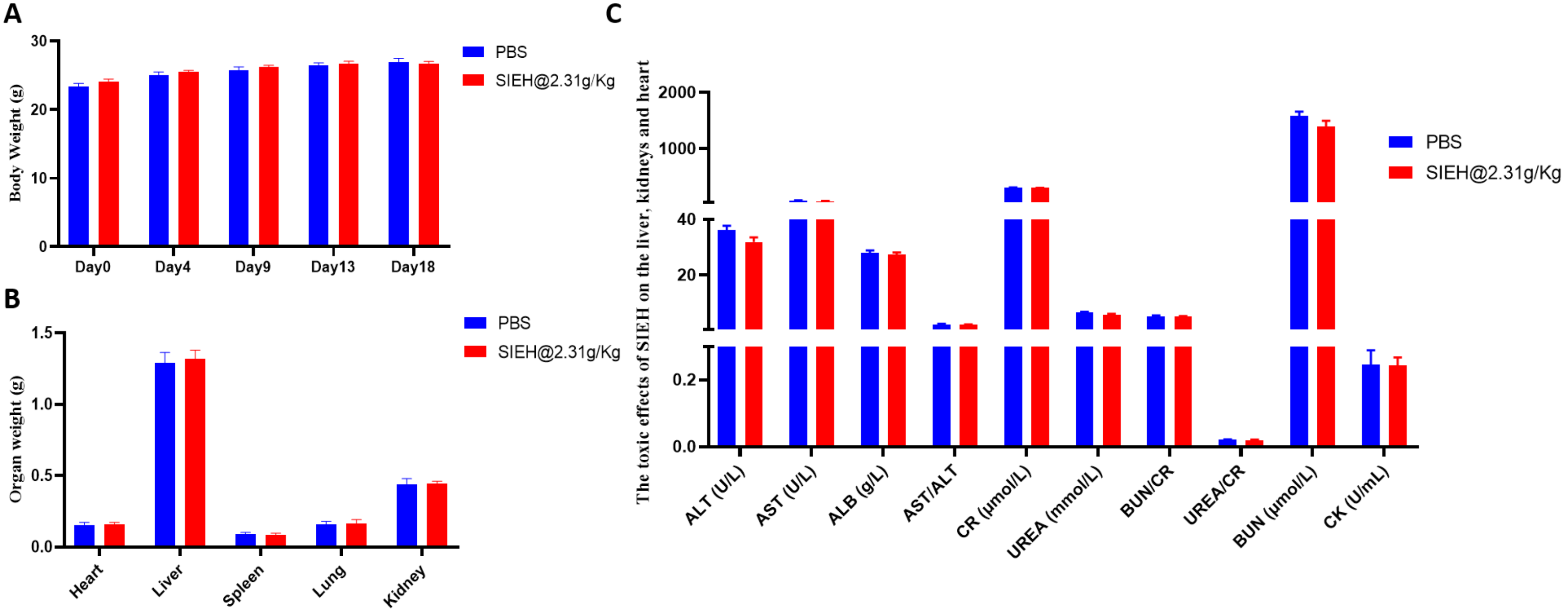

Figure S2. Multi-parameter evaluation of the toxicological effects of SIEH on mice. (A) Body weight; (B) Organ weight; (C) The toxic effect of SIEH on Liver, Kidney and Heart. ALT (U/L), AST (U/L), ALB (g/L) and AST/ALT are liver toxicity markers; CR (μmol/L), UREA (mmol/L), BUN (μmol/L), BUN/CR and UREA/CR are kidney toxicity markers; CK (U/mL) is heart toxicity marker. Group comparisons were performed using T-test and Mann-Whitney U test. Data are expressed as mean ± standard error of mean (n=8 per group). Compared to the PBS group, $^{*}P < 0.05$, $^{**}P < 0.01$. Eight-week-old male ICR mice were used for the experiment. The mice were divided into two groups, a vehicle control group (PBS) and an SIEH group, with 8 mice in each group. The treatments were administered by oral gavage for 18 days. Acute toxicity studies demonstrated that SIEH exhibited no significant toxicity in mice at 2.31g/kg as evidenced by normal body weight gain, absence of organ weight alterations, and unremarkable serum biochemistry parameters.
